# Reactivity of Cyclopropenylaluminates

**DOI:** 10.1021/acs.organomet.5c00272

**Published:** 2025-08-27

**Authors:** Marco F. Starostzik, Jakub Kenar, Han-Ying Liu, Mary F. Mahon, Michael S. Hill

**Affiliations:** Department of Chemistry, 1555 University of Bath, Claverton Down, Bath BA2 7AY, U.K.

## Abstract

Reactions of the potassium cyclopropenylaluminates, [{SiN^Dipp^}­Al-η^2^-(C_2_Ph_2_)­K] and [{SiN^Dipp^}­Al-η^2^-(PhC=CSiMe_3_)­K] with terminal alkynes provide alkynylvinylaluminate derivatives with the silyl-substituted analog providing a level of kinetic discrimination. While this latter behavior results in the regiochemical protonation at Al–C­(Ph) and retention of the more sterically congested Al–C­(SiMe_3_) bond, reactions with CO_2_ and phenyl-substituted ketones are complicated by a reduced level of discrimination and a likely tendency toward multiple C=O insertion or loss of coordinated alkyne. This latter process results in reactivity more reminiscent of the Al­(I) compounds used to synthesize the cyclopropenylaluminate starting materials. Similar observations are provided by reactions with organic azides and trimethylsilyldiazomethane, which proceed with terminal nitrogen insertion and the generation of azacyclobutenylaluminate structures for [{SiN^Dipp^}­Al-η^2^-(C_2_Ph_2_)­K], but with evidence of greater degrees of competitive alkyne elimination from [{SiN^Dipp^}­Al-η^2^-(PhC=CSiMe_3_)­K].

## Graphical abstract

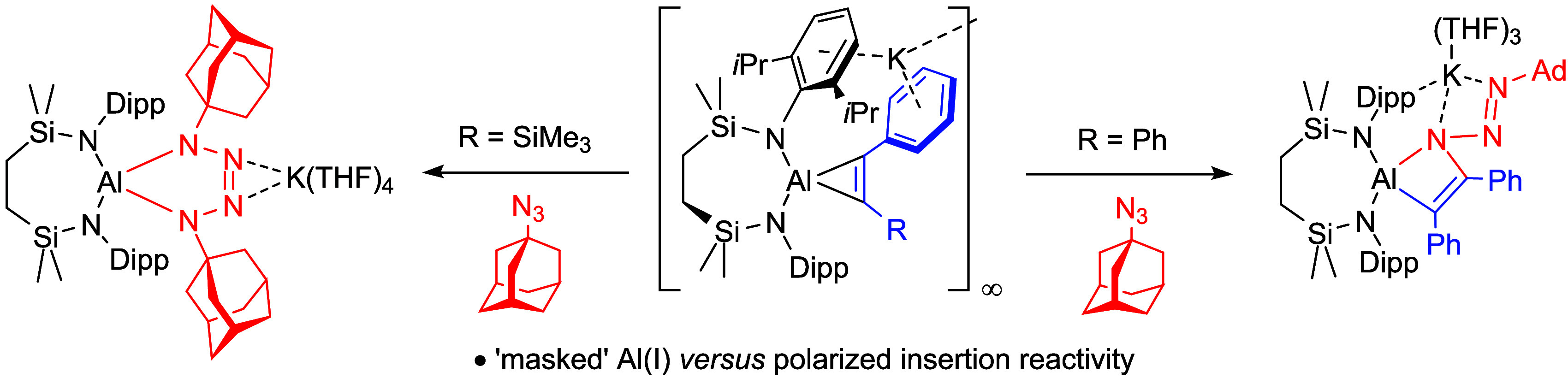

## Introduction

While predated by Uhl’s product of Al–Al insertion from the reaction of tetrakis­[bis­(trimethylsilyl)­methyl]­dialane(4) with lithium phenylethynide,^1^ access to three-membered AlC_2_ heterocycles has more commonly been achieved by oxidation of molecular Al­(I) species. Such compounds engage by effective [2 + 1] cycloaddition with the unsaturated C–C bonds of alkenes and alkynes.^2,3^ For example, β-diketiminate (BDI = HC­{C­(Me)­NDipp}_2_; Dipp = 2,6-*i*-Pr_2_C_6_H_3_) derivatives such as **I**,^4,5^ react with C=C and C≡C containing molecules to provide the respective aluminacyclopropane and aluminacyclopropene derivatives (Scheme 1).^6–10^

**Scheme 1:**
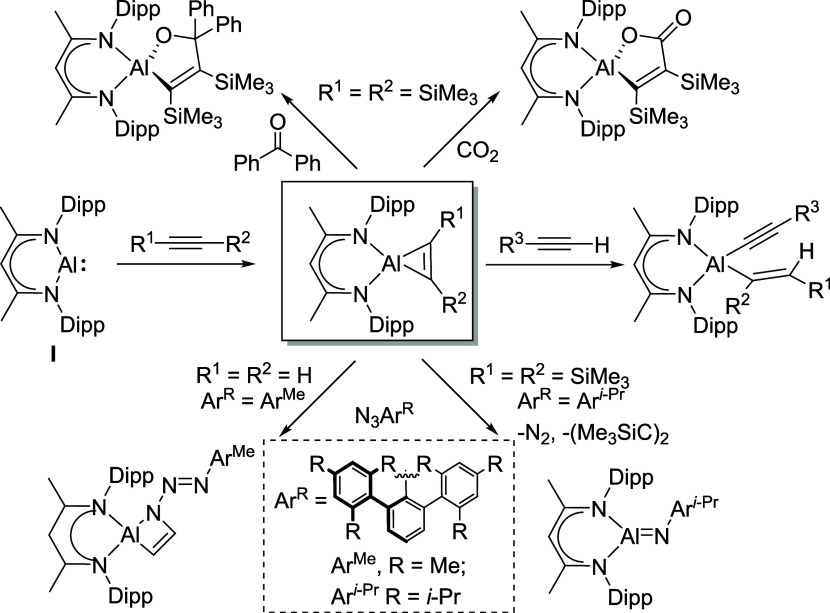
Synthesis of Aluminacyclopropenes from **I** and Their Reactivity with Various Molecular Substrates

In common with wholly organic analogues (e.g., cyclopropanes, cyclopropenes, aziridines, epoxides), the ring strain intrinsic to the resultant aluminacyclo-propane or -propene species has been identified as a potential source of useful and/or unusual reactivity. Acetylene and terminal alkynes, for example, act as a source of proton toward the AlC_2_ unit of [(BDI)­Al­{η^2^-C_2_(R^1^)­(R^2^)}] to induce ring opening and the generation of a range of alkenylalkynylaluminums, [(BDI)­Al­(CR^2^=CR­(H)^1^)­(C≡CR^3^)] (R^1^ = R^2^ = R^3^ = H; R^1^ = H, R^2^ = R^3^ = Ph; R^1^ = R^2^ = Ph, R^3^ = H; R^1^ = R^2^ = R^3^ = Ph; Scheme 1).^7,8^ The analogous product of bis­(trimethylsilyl)­acetylene addition to **I**, [(BDI)­Al­{η^2^-C_2_(SiMe_3_)_2_}], albeit prepared by *in situ* reduction of [(BDI)­AlI_2_] in the presence of the alkyne,^6^ was shown to insert the unsaturated molecules Ph_2_CO and PhCN into one the Al–C bonds providing a novel means of C–C bond formation and the ring-expanded products [(BDI)­Al­{[OC­(O)­C_2_(SiMe_3_)_2_}]], [(BDI)­Al­{OC­(Ph)_2_C_2_(SiMe_3_)_2_}], and [(BDI)­Al­{NC­(Ph)­C_2_(SiMe_3_)_2_}] (Scheme 1).

Analogous insertive reactivity was observed between [(BDI)­Al­{η^2^-C_2_(SiMe_3_)_2_}] and CO, *tert*-butyl isocyanide and CO_2_ (Scheme 1),^11^ while CS_2_ addition was accompanied by C–S bond cleavage and formation of a seven-membered allenylaluminum sulfur heterocycle, [{(BDI)­Al}_2_(μ-S)­{η^2^-SC­(SiMe_3_)=C=C­(SiMe_3_)}].^12^ Treatment of [(BDI)­Al­{η^2^-C_2_(SiMe_3_)_2_}] with pyridine resulted in nucleophilic dearomatization of the nitrogen heterocycle and the production of a (1,2-dihydropyridyl)aluminum species,^13^ while addition of the ethyne-derived analogue, [(BDI)­Al­{η^2^-C_2_H_2_}], to a sterically encumbered azide, Ar^Me^N_3_ (Ar^Me^ = 2,6-(2,4,6-Me_3_-C_6_H_2_)_2_C_6_H_3_), provided C–N bond formation through insertion of the terminal nitrogen to yield a 4-membered aluminaazacyclobutane, [(BDI)­Al­{CH=CHN­(N=NR)}] (Scheme 1).^7^ In contrast, reactions of the more sterically encumbered [(BDI)­Al­{η^2^-C_2_(SiMe_3_)_2_}] with Ar^*i*‑Pr^N_3_ (Ar^*i*‑Pr^ = 2,6-(2,4,6-*i*-Pr_3_–C_6_H_2_)_2_C_6_H_3_) or Ph_3_SiN_3_ resulted in elimination of bis­(trimethylsilyl)­acetylene and the spectroscopic identification of the neutral imides, [(BDI)­Al=NR] (R = Ar^*i*‑Pr^ or Ph_3_Si).^6^ This contrasting outcome implies that such aluminacyclopropenyl derivatives also hold the potential to act as alkyne-“masked” surrogates for the Al­(I) center of **I** (Scheme 1).^6^

A more recent focal point for the Al­(I) oxidation state has been provided by anionic “alumanyl” derivatives (e.g., the anions **II**–**V**, Figure 1a).^3,14–19^ Although the potassium alumanyl (**II** ^**K**^) was only described in 2018, a rich chemistry has emerged.^20–22^ The groups of Aldridge and Coles have, for example, reported that potassium alumanyls, **II** ^**K**^ and **III** ^**K**^, react with ethene under mild conditions (1 bar, room temperature) to provide the corresponding aluminacyclopropanes,^23^ which further provide unusual substrates for onward C–C bond formation with CO (Figure 1b),^23c^ CO_2_ and *i-*PrN=C=N*i*-Pr.^24^ In a similar manner, Yamashita and co-workers have reported that the dialkyl-substituted variant **V** ^**K**^ provides the cyclopropenylaluminate product with diphenylacetylene.^23b^ Although the dimeric potassium alumanyl, [{SiN^Dipp^}­AlK]_2_ (SiN^Dipp^ = {CH_2_SiMe_2_NDipp}_2_^2–^; **IV** ^**K**^), reacts similarly with two equivalents of RC≡CR′ (R = R′ = Ph; R = Ph, R′ = SiMe_3_) to provide **VI** and **VII** (Figure 1c), use of a 1:1 stoichiometry of the dimeric alumanyl and alkyne reagents can also result in 2-fold functionalization at both the C≡C triple bond and a *para*-methine C–H bond of the phenylalkyne units.^25^ While this latter transformation was suggested to take place sequentially and to be an allosteric consequence of the dimeric structure of **IV** ^**K**^, in this contribution, we continue our study of the potassium alkynylaluminate species, **VI** and **VII**, and their reactivity toward terminal alkynes and unsaturated small molecules.

**Figure 1:**
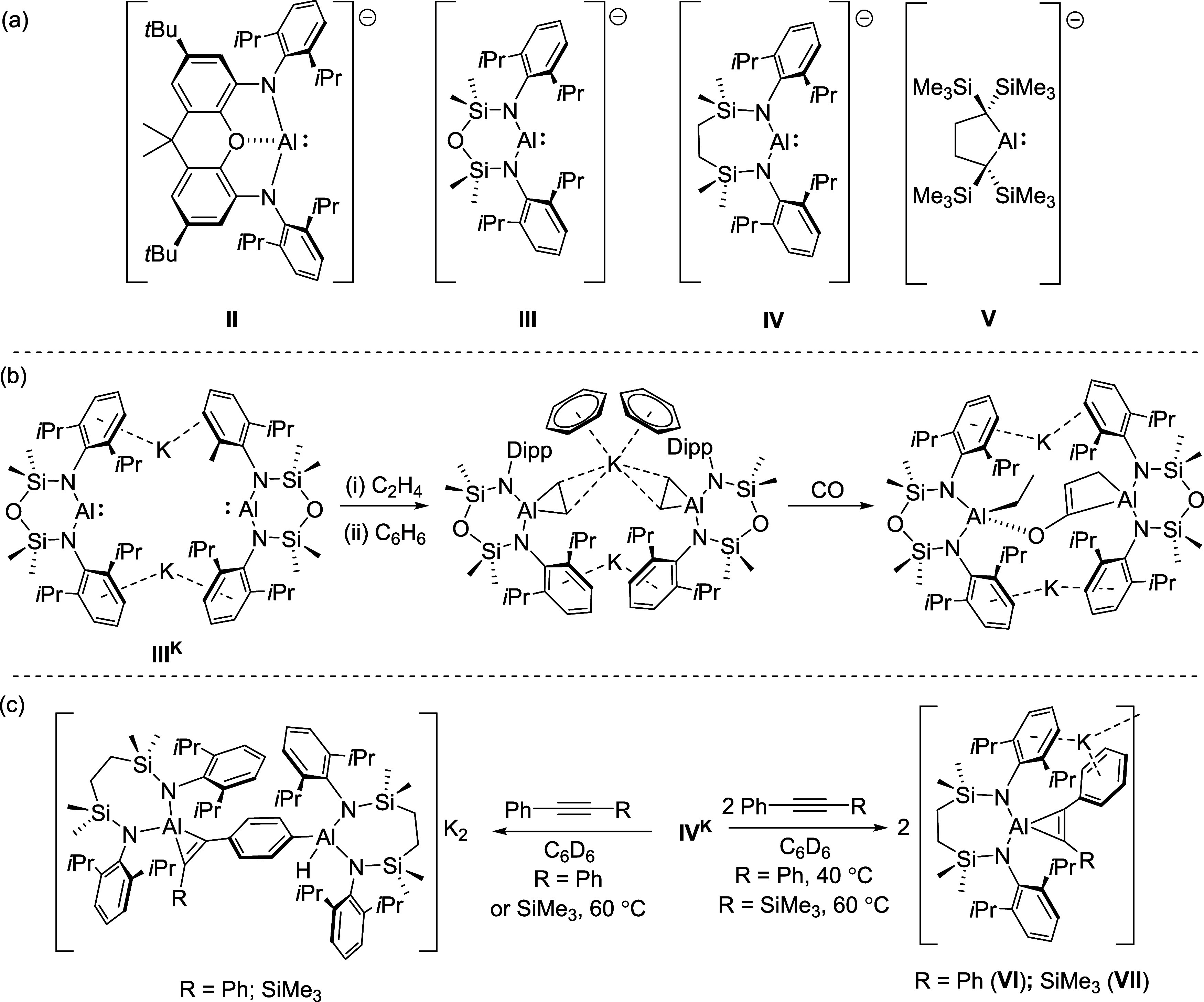
(a) The structures of the anions of compounds **II**–**V**; (b) the sequential reactivity of **III** ^**K**^ with ethylene and CO; (c) the reactivity of **IV** ^**K**^ with phenyl-substituted internal alkynes.

## Experimental Section

### General Considerations

Unless stated otherwise, all experiments were conducted using standard Schlenk line and/or glovebox techniques under an inert atmosphere of argon. NMR spectra were recorded with a Bruker Avance III spectrometer (^1^H at 400 MHz, ^13^C at 101 MHz) or an Agilent ProPulse spectrometer (^1^H at 500 MHz, ^13^C at 126 MHz). The spectra are referenced relative to residual protio solvent resonances. Elemental analyses were performed at Elemental Microanalysis Ltd., Okehampton, Devon, UK. Solvents were dried by passage through a commercially available solvent purification system and stored under argon in ampules over 4 Å molecular sieves. Benzene-*d*_6_ and THF-*d*_8_ were purchased from Sigma-Aldrich and dried over a potassium mirror before distillation and storage over molecular sieves. Compounds **IV** ^**K**^, **VI** and **VII** were prepared according to reported procedures.^15,25^ The terminal acetylenes were purchased from Merck and degassed by three freeze–pump–thaw cycles and stored over 4 Å molecular sieves for more than 18 h prior to usage. Other chemicals were purchased from Merck and used without further purification.

#### Synthesis of [{SiN^Dipp^}­Al­{C­(Ph)=C­(H)­(Ph)}­(C≡CPh)]­K (**1**)

A C_6_D_6_ solution of [{SiN^Dipp^}­Al-η^2^-(C_2_Ph_2_)­K] (**VI**, 37 mg, 0.05 mmol) was charged into a J Young’s NMR tube. Phenylacetylene (5.1 mg, 5.50 μL, 0.05 mmol) was then added to the orange mixture *via* a micropipette. The reaction mixture was kept at ambient temperature for 14 days, during which time the reaction mixture transformed into a colorless solution with complete consumption of the starting materials indicated by ^1^H NMR spectroscopy. An identical outcome was achieved by keeping the same reaction mixture at 40 °C for 3 days. Colorless single crystals of **1** suitable for X-ray diffraction analysis were obtained by adding one drop of THF to the crude reaction mixture and allowing the clear solution to slowly evaporate at ambient temperature for 2 weeks. Yield: 36.0 mg, 68%. Anal. Cal’d. for C_64_H_90_AlKN_2_O_3_Si_2_ (**1**.(OC_4_H_8_)_3_): C, 72.68%; H, 8.58%; N, 2.65%. Found: C, 72.24%; H, 8.46%; N, 2.83%. ^1^H NMR (400 MHz, 298 K, *d*_8_-THF): δ 6.88 (d_app_, 4H, Ar*H*), 6.82–6.80 (m, 2H, Ar*H*), 6.76 (dd_app_, 2H, Ar*H*), 6.71 (dd_app_, 2H, Ar*H*), 6.67–6.63 (m, 4H, Ar*H*), 6.59–6.56 (m, 3H, Ar*H*), 6.52–6.44 (m, 3H, Ar*H*), 5.94 (d, 2H, *o*-C_6_ *H*_5_), 4.37 (sept, *J* = 6.7 Hz, 2H, C*H*Me_2_), 4.20 (sept, *J* = 6.7 Hz, 2H, C*H*Me_2_), 1.06 (d, *J* = 6.7 Hz, 6H, CH*Me*_2_), 1.00 (d, *J* = 6.7 Hz, 6H, CH*Me*_2_), 0.97^+^ (d, 6H, CH*Me*_2_), 0.96^+^ (s, 4H, SiC*H*_2_), ^+^overlapping peaks, 0.79^#^ (d, 3H, CH*Me*_2_), δ 0.78^#^ (d, 3H, CH*Me*_2_), ^#^overlapping peaks, δ 0.00 (s, 6H, Si*Me*_2_), −0.05 (s, 6H, Si*Me*_2_). ^13^C­{^1^H}­NMR (101 MHz, 298 K, *d*_8_-THF): δ 152.3 (Al–C≡*C*), 152.0 (*i*-*C*_6_H_5_), 148.8 (*i*-*C*_6_H_3_), 147.9 (*o*-*C*_6_H_3_), 136.9 (*i*-*C*_6_H_5_), 132.4 (*o*-*C*_6_H_5_), 130.2 (*i*-*C*_6_H_5_), 130.1 (Al–C=*C*), 127.9 (*o*-*C*_6_H_5_), 127.8 (Ar*C*), 127.5 (Ar*C*), 127.0 (Ar*C*), 125.1 (Ar*C*), 124.3 (Ar*C*), 124.1 (*m*-*C*_6_H_3_), 123.3 (*p*-*C*_6_H_3_), 121.9 (Ar*C*), 121.4 (Ar*C*), 28.1 (*C*HMe_2_), 27.9 (*C*HMe_2_), 27.5 (CH*Me*_2_), 27.3 (CH*Me*_2_), 27.1 (CH*Me*_2_), 26.7 (CH*Me*_2_), 26.6 (CH*Me*_2_), 16.0 (Si*C*H_2_), 3.7 (Si*Me*_2_), 3.1 (Si*Me*_2_). ^13^C­{^1^H} resonances correlated to Al*C*=C and Al-*C*≡C were not observed.

#### Synthesis of [{SiN^Dipp^}­Al­{C­(SiMe_3_)=C­(H)­(Ph)}­(C≡CPh)]­K (**2**)

Phenylacetylene (5.1 mg, 5.5 μL, 0.05 mmol) was added to a C_6_D_6_ solution of [{SiN^Dipp^}­Al-η^2^-*C*,*C*’-(PhCCSiMe_3_)­K] (**VII**, 36.8 mg, 0.05 mmol) *via* a micropipette. The reaction mixture was kept at room temperature for 1 week, whereupon a gradual decolorisation of the dark red solution was observed. The crude reaction mixture was inferred by ^1^H NMR spectroscopy to have transformed quantitatively into a single new compound (**2**). Single crystals suitable for X-ray diffraction analysis were obtained by slow evaporation of a benzene solution. The colorless crystals were collected and washed with *n*-hexane (3 × 0.3 mL) before the removal of all volatiles *in vacuo*, to afford **2** as a colorless powder. Yield: 30.1 mg, 72%. Anal. Cal’d. for C_57_H_86_AlKN_2_O_2_Si_3_ (**2**-(OC_4_H_8_)_2_, 981.66): C, 69.74%; H, 8.83%; N, 2.85%. Found: C, 69.68%; H, 8.96%; N, 2.88%. ^1^H NMR (400 MHz, 298 K, *d*_8_-THF) δ 7.77 (s, 1H, AlC­(SiMe_3_)­C­(Ph)­(*H*)), 7.21–7.15 (m, 2H, *o*-C≡C-*Ph*), 7.08–7.01 (m, 3H, *m, p*-C­(SiMe_3_)­C­(*Ph*)­(H)), 6.98–6.91 (m, 3H, *m-, p-*C≡C-*Ph*), 6.86 (dd, *J* = 7.5, 1.9 Hz, 2H, *m*-C_6_ *H*_3_), 6.82 (m, 2H, *o*-C­(SiMe_3_)­C­(*Ph*)­(H)), 6.77 (dd, *J* = 7.6, 1.9 Hz, 2H, *m-*C_6_ *H*_3_), 6.67 (t, *J* = 7.5 Hz, 2H, *p*-C_6_ *H*_3_), 4.55 (sept, *J* = 6.6 Hz, 2H, C*H*Me_2_), 4.22 (sept, *J* = 6.6 Hz, 2H, C*H*Me_2_), 1.30 (d, *J* = 6.6 Hz, 6H, CH*Me*_2_), 1.16* (d, *J* = 6.6 Hz, 8H, CH*Me*_2_ and SiC*H*_2_), 1.12 (d, *J* = 6.6 Hz, 6H, CH*Me*_2_), 1.05* (d, *J* = 6.6 Hz, 8H, CH*Me*_2_ and SiC*H*_2_), 0.19 (s, 6H, Si*Me*_2_), −0.06 (s, 6H, Si*Me*_2_), −0.50 (s, 9H, C­(Si*Me*_3_)­C­(Ph)­(H)).*overlapping peaks; ^13^C­{^1^H} NMR (101 MHz, 298 K, *d*_8_-THF) δ 155.8 (C­(SiMe_3_)*C*(Ph)­(H)), 152.6 (*i*-*C*_6_H_3_), 148.9 (*o*-*C*_6_H_3_), 147.7 (*o-C*_6_H_3_), 132.1 (*o*-C≡C-*C*_6_H_5_), 130.6 (*i*-C=C-*C*_6_H_5_), 129.5 (*o*-C=C-*C*_6_H_5_), 128.0 (*m*-C≡C-*C*_6_H_5_), 127.1 (*m*-C=C-*C*_6_H_5_), 125.2 (*p*-C = C-*C*_6_H_5_), 125.1 (*p*-C≡C-*C*_6_H_5_), 123.8 (*m*-*C*_6_H_3_), 123.3 (*m*-*C*_6_H_3_), 121.4 (*p*-*C*_6_H_3_), 105.9* (C≡*C*-C_6_H_5_), 28.2 (*C*HMe_2_), 27.9 (*C*HMe_2_), 27.5 (CH*Me*_2_), 27.2 (CH*Me*_2_), 27.0 (CH*Me*_2_), 26.9 (CH*Me*_2_), 24.3 (Si*C*H_2_), 16.4 (Si*C*H_2_), 4.4 (Si*Me*_2_), 4.2 (Si*Me*_2_), 4.1 (Si*Me*_3_).*seen in 2D NMR. ^13^C resonances correlated to Al-*C*=C Al*-C*≡C were not observed.

#### Synthesis of [{SiN^Dipp^}­Al­{C­(SiMe_3_)=C­(H)­(Ph)}­(C≡CSiMe_3_)]K (**3**)

Me_3_SiCCH (4.9 mg, 6.9 μL, 0.05 mmol) was added to a C_6_D_6_ solution of [{SiN^Dipp^}­Al-η^2^-C,C’-(PhCCSiMe_3_)­K] (**VII**, 36.8 mg, 0.05 mmol) *via* a micropipette. The reaction mixture was stored at room temperature for 2 weeks, whereupon a gradual decolorisation of the dark red solution was observed. Analysis of the crude reaction mixture by ^1^H NMR spectroscopy, implied the quantitative formation of a single compound (**3**). A single crystal suitable for X-ray diffraction analysis was obtained by slow evaporation of a THF/hexane solution. Colorless crystals were collected and washed with *n*-hexane (3 × 0.3 mL) before the removal of all volatiles *in vacuo*, to afford **3** as a colorless powder. Yield: 28.8 mg, 69%. No meaningful elemental analysis was obtained after multiple attempts. ^1^H NMR (400 MHz, 298 K, *d*_8_-THF) δ 7.69 (s, 1H, AlC­(SiMe_3_)­C­(Ph)*H*), 7.01–6.90 (m, 2H, C_6_ *H*_5_), 6.86 (dd, *J* = 7.5, 1.9 Hz, 2H, *m*-C_6_ *H*_3_), 6.84–6.80 (m, 3H, C_6_ *H*_5_), 6.76 (dd, *J* = 7.5, 1.9 Hz, 2H, *m*-C_6_ *H*_3_), 6.66 (t, *J* = 7.5 Hz, 2H, *p*-C_6_ *H*_3_), 4.47 (sept, *J* = 6.9 Hz, 2H, C*H*Me_2_), 4.15 (sept, *J* = 6.9 Hz, 2H, C*H*Me_2_), 1.36 (d, *J* = 6.9 Hz, 6H, CH*Me*_2_), 1.17 (d, *J* = 6.9 Hz, 6H, CH*Me*_2_), 1.13–1.06 (m, 8H, CH*Me*_2_ and SiC*H*_2_), 1.05–0.97 (m, 8H, CH*Me*_2_ and SiC*H*_2_), 0.18 (s, 6H, Si*Me*_2_), −0.05 (s, 9H, Si*Me*_3_), −0.15 (s, 6H, Si*Me*_2_), −0.51 (s, 9H, Si*Me*_3_); ^13^C­{^1^H} NMR (101 MHz, 298 K *d*_8_-THF) δ 155.7 (AlC­(SiMe_3_)*C*(Ph)­H), 152.6 (*i*-*C*_6_H_3_), 148.8 (*o*-*C*_6_H_3_), 147.7 (*o*-*C*_6_H_3_), 129.5 (*C*_6_H_5_), 127.1 (*C*_6_H_5_), 125.1 (*C*_6_H_3_), 123.9 (*m*-*C*_6_H_3_), 123.2 (*m*-*C*_6_H_3_), 121.4 (*p-C*_6_H_3_), 101.3* (C≡*C*-C_6_H_5_) *seen in 2D, 28.0 (*C*HMe_2_), 27.6 (*C*HMe_2_), 27.1 (CH*Me*_2_), 27.0 (CH*Me*_2_), 26.9 (CH*Me*_2_), 26.6 (CH*Me*_2_), 16.2 (Si*C*H_2_), 4.7 (Si*Me*_2_), 4.6 (Si*Me*_3_), 4.3 (Si*Me*_2_), 1.3 (Si*Me*_3_). ^13^C resonances correlated to Al-*C*=C and Al-*C*≡C were not observed.

#### Synthesis of [{SiN^Dipp^}­Al-κ^2^-*C*,*O*-(C­(Ph)=C­(Ph)–C­(O)-O)]­K (**4**)

A C_6_D_6_ solution of [{SiN^Dipp^}­Al-η^2^-(C_2_Ph_2_)­K] (**VI**, 37 mg, 0.05 mmol) was degassed by three freeze–pump–thaw cycles before being charged with 2 atm. of ^13^CO_2_. The resultant ^1^H and ^13^C­{^1^H} NMR spectra indicated instantaneous consumption of the starting materials. All volatiles were then removed *in vacuo*, affording a pale yellow waxy solid. Colorless single crystals suitable for X-ray diffraction analysis were grown from a hexane solution with one drop of THF. Yield: 32.5 mg, 76%. No meaningful elemental analysis was obtained after multiple attempts. ^1^H NMR (400 MHz, 298 K, *d*_8_-THF): δ 6.91–6.88 (m, 2H, *o*-C_6_ *H*_5_), 6.79–6.59 (m, 12H, Ar*H*), 6.09–6.06 (m, 2H, C_6_ *H*_5_), 4.16 (sept, *J* = 6.7 Hz, 2H, C*H*Me_2_), 4.01 (sept, *J* = 6.7 Hz, 2H, C*H*Me_2_), 3.63–3.58 (m, 4H, coordinated THF), 1.79–1.74 (m, 4H, coordinated THF), 1.36 (d, *J* = 6.7 Hz, 6H, CH*Me*_2_), 1.15 (d, *J* = 6.7 Hz, 6H, CH*Me*_2_), 1.12 (d, *J* = 6.7 Hz, 6H, CH*Me*_2_), 1.01 (s, 4H, SiC*H*_2_), 0.65 (d, *J* = 6.7 Hz, 6H, CH*Me*_2_), 0.00 (s, 6H, Si*Me*_2_), −0.02 (s, 6H, Si*Me*_2_). ^13^C­{^1^H}­NMR (101 MHz, 298 K, *d*_8_-THF): δ 175.4 (O_2_ *C*), 164.9 (O_2_C-*C*=C) 149.6 (*i*-*C*_6_H_5_), 147.8 (*i*-*C*_6_H_3_), 147.2 (*o*-*C*_6_H_3_), 131.9 (*i-C*_6_H_5_), 127.7 (Ar*C*), 126.8 (Ar*C*), 126.6 (Ar*C*), 125.3 (Ar*C*), 124.0 (Ar*C*), 123.7 (Ar*C* on *C*_6_H_5_), 123.4 (Ar*C* on *C*_6_H_3_), 122.1 (*o-C*_6_H_5_), 28.0 (*C*HMe_2_), 27.6 (*C*HMe_2_), 26.4 (CH*Me*_2_), 26.3 (CH*Me*_2_), 26.2 (CH*Me*_2_), 26.1 (CH*Me*_2_), 15.5 (Si*C*H_2_), 2.4 (Si*Me*_2_), 2.1 (Si*Me*_2_).

#### Synthesis of [{SiN^Dipp^}­Al-κ^2^-*C*,*N*-{C­(Ph)=C­(Ph)-N­(NCHSiMe_3_)}]K (**6**)

Acetophenone (6 mg, 5.82 μL, 0.05 mmol) was added to an orange C_6_D_6_ solution of [{SiN^Dipp^}­Al-η^2^-(C_2_Ph_2_)­K] (**VI**, 37 mg, 0.05 mmol). The crude mixture was then left at ambient temperature for 1 day, forming a colorless precipitate with a pale pink supernatant. The precipitate was collected and dissolved as a THF/toluene/hexane solution (5 drops/0.1 mL/0.5 mL). Colorless single crystals suitable for X-ray diffraction analysis were obtained after storage at ambient temperature. Yield: 29 mg, 62%. No meaningful elemental analysis was obtained after multiple attempts. ^1^H NMR (400 MHz, 298 K, *d*_8_-THF): δ 7.11 (s br, 2H, Ar*H*), 6.92–6.90 (dd_app_, 1H, *m*-C_6_ *H*_5_), 6.87–6.85 (dd_app_, 1H, *m*-C_6_ *H*_5_), 6.82–6.81 (dd_app_, 1H, *m*-C_6_ *H*_5_), 6.77 (t, 3H, Ar*H*), 6.72–6.65 (m, 5H, Ar*H*), 6.57–6.55 (dd_app_, 3H, Ar*H*), 6.39 (t, 2H, *p*-C_6_ *H*_5_), 6.26 (t, 1H, Ar*H*), 6.05 (s br, 2H, Ar*H*), 4.49 (sept, *J* = 6.7 Hz, 1H, C*H*Me_2_), 4.17–4.06 (3 x sept_app_, 3H, C*H*Me_2_), 1.48 (d, *J* = 6.7 Hz, 3H, CH*Me*_2_),1.44 (d, *J* = 6.7 Hz, 3H, CH*Me*_2_), 1.25–1.22 (m, 6H, CH*Me*_2_), 1.16 (s, 1H, SiC*H*_2_), 1.15 (s, 1H, SiC*H*_2_), 1.10 (d, J = 6.7 Hz, 3H, CH*Me*_2_), 1.01 (d, J = 6.7 Hz, 3H, CH*Me*_2_), 0.95 (s, 1H, SiC*H*_2_), 0.57 (s, 3H, Si*Me*_2_), 0.49 (d, *J* = 6.7 Hz, 3H, CH*Me*_2_), 0.44 (s, 3H, OC*Me*), 0.34 (s, 3H, Si*Me*_2_), 0.21 (d, *J* = 6.7 Hz, 3H, CH*Me*_2_), 0.06 (s, 1H, SiC*H*_2_), −0.44 (s, 3H, Si*Me*_2_), −0.46 (s, 3H, Si*Me*_2_). ^13^C­{^1^H} NMR (101 MHz, 298 K, *d*_8_-THF): δ 167.6 (*i*-*C*_6_H_5_), 165.9 (*i*-*C*_6_H_5_), 160.4 (*i*-*C*_6_H_3_), 157.0 (*i*-*C*_6_H_3_), 155.8 (*i*-*C*_6_H_3_), 153.6 (*i*-*C*_6_H_3_), 152.5 (*i*-*C*_6_H_3_), 151.0 (*i-C*_6_H_3_), 133.0 (Ar*C*), 132.3 (Ar*C*), 132.2 (Ar*C*), 130.6 (Ar*C*), δ 130.5 (Ar*C*), δ 130.4 (Ar*C*), δ 128.5 (Ar*C*), δ 128.4 (Ar*C*), 128.3 (Ar*C*), 127.3 (Ar*C*), 125.6 (Ar*C*), 125.5 (Ar*C*), 125.1 (ArC), 85.5 (O*C*), 38.0 (OC*Me*), 32.7 (*C*HMe_2_), 32.6 (*C*HMe_2_), 32.0 (CH*Me*_2_), 31.4 (CH*Me*_2_), 31.3 (CH*Me*_2_), 31.2 (CH*Me*_2_), 31.1 (CH*Me*_2_), 30.8 (CH*M*e_2_), 30.7 (CH*Me*_2_), 28.5 (CH*Me*_2_), 19.6 (Si*C*H_2_), 19.5 (Si*C*H_2_), 9.6 (Si*Me*_2_), 8.4 (Si*Me*_2_), 7.6 (Si*Me*_2_).

#### Synthesis of [{SiN^Dipp^}­Al-κ^2^-*C*,*N*-{C­(Ph)=C­(Ph)-N­(N_2_Ad)}]K (**7**)

1-azidoadamantane, (AdN_3_, 8.9 mg, 0.05 mmol) was added to a C_6_D_6_ solution of [{SiN^Dipp^}­Al-η^2^-(C_2_Ph_2_)­K] (**VI**, 37 mg, 0.05 mmol), whereupon the reaction mixture displayed an instantaneous color change from bright yellow to colorless. Single crystals suitable for X-ray diffraction analysis were obtained by slow evaporation of the C_6_D_6_ solution. The colorless crystals were washed with *n*-hexane (3 × 0.3 mL) before the removal of all volatiles *in vacuo*, to afford **7** as a colorless powder. Yield: 29.8 mg, 65%. Anal. Calc’d. for C_69_H_90_AlKN_5_Si_2_ (i.e., **7-**(2.5C_6_H_6_), 1072.58): C, 73.90%; H, 8.17%; N, 6.53%. Found, C, 74.34%; H, 8.46%; N, 5.98%. ^1^H NMR (400 MHz, 298 K, *d*_8_-THF): δ 7.00–6.95 (m, 2H, *o-*N–C–C_6_ *H*_5_), 6.89 (dd, *J* = 7.5, 1.8 Hz, 2H, *m*-C_6_ *H*_3_), 6.80 (dd, *J =* 7.5, 1.8 Hz, 2H, *m*-C_6_ *H*_3_), 6.73 (t, *J* = 7.5 Hz, 2H, *p*-C_6_ *H*_3_), 6.71–6.63 (m, 3H, *m,p-*N–C-*Ph*), 6.45 (t, *J* = 7.2 Hz, 2H, *m-*Al–C-*Ph*), 6.39–6.34 (m, 1H, *p-*Al–C-*Ph*), 5.56 (d, *J* = 7.2 Hz, 2H, *o-*Al–C-*Ph*), 3.98* (sept, *J* = 6.7 Hz, 2H, C*H*Me_2_), 3.93* (sept, *J* = 6.7 Hz, 2H, C*H*Me_2_) 2.01 (br. s, 3H, C*H* on Ad), 1.85 (d, *J* = 3.0 Hz, 6H, C*H*_2_ on Ad), 1.67 (d, *J* = 3.0 Hz, 6H, C*H*_2_ on Ad), 1.44–1.33 (m, 4H, SiC*H*_2_), 1.15–1.06 (m, 18H, CH*Me*_2_), 0.66 (d, *J* = 6.7 Hz, 6H, CH*Me*_2_), 0.29 (s, 6H, Si*Me*_2_), −0.11 (s, 6H, Si*Me*_2_). *Overlapping peaks ^13^C­{^1^H} NMR (101 MHz, 298 K, *d*_8_ *-*THF) δ 157.7 (N-*C*), 149.9 (*i*-*C*_6_H_3_), 148.9 (*o*-*C*_6_H_3_), 147.2 (*o*-*C*_6_H_3_), 137.2 (*i*–Al-C-*C*_6_H_5_), 132.7 (*o*–N-C-*Ph*), 129.9, 126.9 (*m-*Al–C-*ph*), 126.0 (*m*–N-C-*Ph*), 124.7 (*p*–N-C-*Ph*), 123.8 (*m*-*C*_6_H_3_), 122.9 (*m*-*C*_6_H_3_), 121.7, (*p*-*C*_6_H_3_), 120.9 (*p*–Al-C-*Ph*), 59.2 (*i*-*C* on Ad), 44.7 (*C*H_2_ on Ad), 38.6 (*C*H_2_ on Ad), 31.6 (*C*H on Ad), 28.4 (*C*HMe_2_), 27.8 (*C*HMe_2_), 27.1 (CH*Me*_2_), 27.1 (CH*Me*_2_), 26.6 (CH*Me*_2_), 25.4 (CH*Me*_2_), 15.6 (Si*C*H_2_), 5.9 (Si*Me*_2_), 3.4 (Si*Me*_2_).

#### Synthesis of [{SiN^Dipp^}­Al-κ^2^-*C*,*N*-{C­(Ph)=C­(Ph)-N­(N_2_C_6_H_2_Me_3_)}]K (**8**)

Mesityl azide (7.3 mg, 0.05 mmol) was added to a C_6_D_6_ solution of [{SiN^Dipp^}­Al-η^2^-(C_2_Ph_2_)­K] (**VI**, 37 mg, 0.05 mmol). After storage of the reaction mixture at ambient temperature for 3 days, an orange precipitate was obtained. The crude mixture was then kept at room temperature for a further 14 days, at which point the supernatant was carefully decanted and the orange solids collected and washed with hexane (2 × 0.2 mL). The solids were then dissolved in a minimal amount of THF and hexane (*c*.*a*. 0.5 mL) was carefully layered on the orange solution. Single crystals suitable for X-ray diffraction analysis were obtained from the layered solution after 5 days at ambient temperature. Yield: 39.5 mg, 71%. No meaningful elemental analysis was obtained after multiple attempts. ^1^H NMR (400 MHz, 298 K, *d*_8_-THF): δ 6.84–6.61 (m, 15H, Ar*H*), 6.47 (s, 2H, Ar*H*), 6.07 (s, 1H, Ar*H*), 6.05 (s, 1H, Ar*H*), 4.16 (2 x sept_app_, 4H, C*H*Me_2_), 2.10 (s, 3H, *p*-C_6_H_2_ *Me*_3_), 2.10 (s, 6H, *o*-C_6_H_2_ *Me*_3_), 1.16–1.07 (m, 24H, CH*Me*_2_), 0.67 (s, 2H, SiC*H*_2_), 0.66 (s, 2H, SiC*H*_2_), 0.16 (s, 6H, Si*Me*_2_), 0.04 (s, 6H, Si*Me*_2_). ^13^C­{^1^H}­NMR (101 MHz, 298 K, *d*_8_-THF): δ 149.3 (*i*-*C*_6_H_5_), 148.4 (*i*-C_6_H_5_), 147.1 (*i*-*C*_6_H_2_Me_3_), 132.5 (Ar*C*), 132.4 (Ar*C)*, 132.3 (Ar*C*), 130.9 (Ar*C*), 130.3 (Ar*C*), 129.3 (Ar*C*), 129.2 (Ar*C*), 128.9 (Ar*C*), 127.1 (Ar*C*), 126.9 (Ar*C*), 123.6 (Ar*C*), 123.2 (Ar*C*), 121.8 (Ar*C*), 28.2 (*p*-C_6_H_2_ *Me*_3_), 27.4 (*o*-C_6_H_2_ *Me*_3_), 27.3 (*C*HMe_2_), 26.6 (*C*HMe_2_), 26.5 (CH*Me*_2_), 26.4 (CH*Me*_2_), 18.3 (CH*Me*_2_), 18.0 (CH*Me*_2_), 20.8 (Si*C*H_2_), 15.8 (Si*C*H_2_), 3.7 (Si*Me*_2_), 3.0 (Si*Me*_2_).

#### Synthesis of [{SiN^Dipp^}­Al-κ^2^-*C*,*N*-{C­(Ph)=C­(Ph)-N­(NCHSiMe_3_)}]K (**10**)

A 2 M solution of trimethylsilyl-diazomethane in hexane (25 μL, 0.05 mmol) was pipetted into an orange C_6_D_6_ solution of [{SiN^Dipp^}­Al-η^2^-(C_2_Ph_2_)­K] (**VI**, 37 mg, 0.05 mmol). The reaction mixture was observed to become pale yellow within 30 min, and a colorless precipitate was obtained after storage at ambient room temperature for 1 day. The solids were collected and dissolved in THF, whereupon slow diffusion of hexane into the resultant solution provided colorless single crystals suitable for X-ray diffraction analysis. Yield, 32 mg, 60%. No meaningful elemental analysis was obtained after multiple attempts. ^1^H NMR (400 MHz, 298 K, *d*_8_-THF): δ 7.02 (dd_app_, 2H, Ar*H*), 6.88–6.86 (m, 3H, Ar*H*), 6.77–6.74 (m, 2H, Ar*H*), 6.71–6.65 (m, 5H, Ar*H*), 6.54–6.50 (m, 2H, Ar*H*), 6.40–6.37 (m, 1H, Ar*H*), 5.91* (s br, 1H, Ar*H*), 5.89* (s br, 1H, *H*CSiMe_3_), *overlapping peaks, 4.08 (sept, *J* = 6.7 Hz, 2H, C*H*Me_2_), 3.95 (sept, *J* = 6.7 Hz, 2H, C*H*Me_2_), 1.19 (d, *J* = 6.7 Hz, 6H, CH*Me*_2_), 1.12+ (d, 12H, CH*Me*_2_), 1.10+ (d, 4H, SiC*H*_2_), + overlapping peaks, 0.65 (d, *J* = 6.7 Hz, 6H, CH*Me*_2_), 0.12 (s, 6H, Si*Me*_2_), 0.01 (s, 6H, Si*Me*_2_), −0.05 (s, 9H, Si*Me*_3_). ^13^C­{^1^H} NMR (101 MHz, 298 K, *d*_8_-THF): δ 149.8 (*i*-*C*_6_H_5_), 148.2 (*i*-*C*_6_H_5_), 147.4 (*i-C*_6_H_3_), 132.8 (Ar*C*), 130.0 (H*C*SiMe_3_), 127.0 (Ar*C*), 125.9 (Ar*C*), 123.5 (Ar*C*), 123.3 (Ar*C*), 121.8 (Ar*C*), 28.4 (*C*HMe_2_), 27.5 (*C*HMe_2_), 27.4 (CH*Me*_2_), 26.9 (CH*Me*_2_), 26.7 (CH*Me*_2_), 15.9 (SiC*H*_2_), 4.3 (Si*Me*_2_), 3.2 (Si*Me*_2_), 0.3 (Si*Me*_3_).

### Reactivity with Terminal Alkynes

An initial study assayed the reactivity of compound **VI** toward PhC≡CH in benzene (Scheme 2). Although the reaction was slow at room temperature, requiring 14 days to achieve complete consumption of the cyclopropenylaluminate starting material, this process was visually apparent by a dissipation in color of the initial deep orange solution. Subsequent experiments determined that a similar outcome could be achieved by heating the reaction mixture to 40 °C for 3 days. Although removal of volatiles at this point provided compound **1** as a colorless solid, its subsequent insolubility in noncoordinating solvents required the addition of THF to a benzene suspension to achieve solubilization. Slow evaporation of the resultant solution provided the bis-THF adduct of **1** as colorless single crystals in moderate to good (68%) yield. Consistent with the loss of the C_2_-symmetric structure of **VI**, analysis of **1** by ^1^H NMR spectroscopy in *d*_8_-THF revealed that the *iso*-propyl methine environments of the {SiN^Dipp^} *N*-aryl substituents presented as a pair of multiplets at δ_H_ 4.37 and 4.20 ppm, each of which resonated as 2H signals by relative integration. X-ray diffraction analysis of **1** performed on a single crystal obtained from a benzene/THF solvent system confirmed that these observations arise from the formation of the potassium alkynido­(vinyl)­aluminate, [{SiN^Dipp^}­Al­{C­(Ph)=C­(H)­(Ph)}­(C≡CPh)]­[K­(THF)_2_] (**1**, Figure 2a). The aluminate component of **1** is, thus, broadly analogous to the, albeit charge neutral, product of terminal alkyne addition to the β-diketiminate species depicted in Scheme 1.

**Figure 2:**
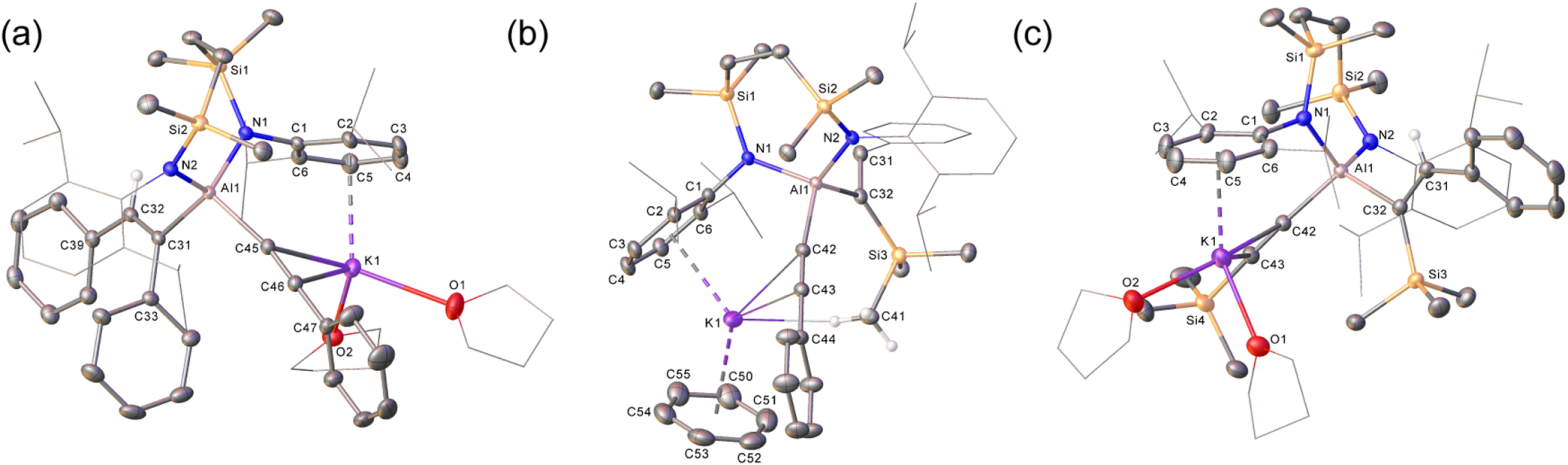
Displacement ellipsoid plots (30% probability) of (a) compound **1**, (b) compound **2** and (c) compound **3**. The majority of hydrogen atoms plus disordered atoms and occluded solvent in **1** and **2** have been removed for clarity. Wireframe view has been employed for some groups, also for visual ease.

**Scheme 2:**
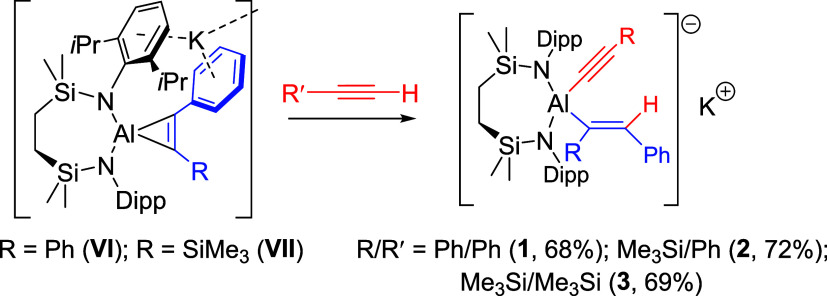
Synthesis of Compounds **1**–**3** (% Indicates Isolated Yield)

In contrast to the reactivity of **VI** and the unambiguous formation of **1**, the less symmetrical aluminacyclopropenyl unit of **VII** provides two differentiated Al–C bonds as potential points of reactivity. A reaction of **VII** with PhC≡CH was, therefore, performed at 40 °C and monitored by ^1^H NMR spectroscopy. Although complete consumption of the starting materials was observed over the course of 1 week, the resultant spectrum was complex and indicative of the formation of a mixture of asymmetric compounds. The emergence of two downfield singlets at δ 8.67 and 7.27 ppm was particularly diagnostic of indiscriminate protonolysis of both the silyl- and phenyl-substituted aluminacyclopropene carbons. Although potassium alkenylalkynylaluminate products could not be isolated by fractional recrystallization, further reactions of **VII** with both phenyl- and trimethylsilylacetylene performed at room temperature over one and 2 weeks, respectively, provided single products. Compounds **2** and **3** were most readily identified from the emergence of vinylic (1H) singlet signals at δ 8.76 (**2**) and 7.69 (**3**) ppm. After removal of volatiles, the low solubility of both compounds in arene solvents necessitated the use of *d*_8_-THF to acquire full solution characterization by NMR spectroscopy. Similarly, although single crystals of **2** could be obtained by slow evaporation of the benzene reaction solution, crystallization of **3** required a mixture of hexane and THF. The outcome of the X-ray diffraction analysis of both compounds is presented in Figure 2b,c with selected bond length and angles in Table 1.

**Table 1:** Selected Bond Lengths (Å) and Angles (°) of Compounds **1**–**3**

	**1**	**2**	**3**
Al1–N1	1.8846(10)	1.8997(10)	1.9083(15)
Al1–N2	1.8742(10)	1.8856(10)	1.8863(14)
Al1–C32	2.0163(11)^a^	2.0256(12)	2.0357(17)
Al1–C42	1.9913(12)^b^	2.0035(12)	2.0091(18)
C31–C32	1.3465(16)	1.3485(18)	1.348(3)
C42–C43	1.2157(17)^c^	1.2134(18)	1.220(3)
N1–Al1–N2	112.01(4)	111.31(4)	110.99(7)
C32–Al1–C42	103.74(5)^d^	110.26(5)	110.32(7)

a Al1–C31.

b Al1–C45.

c C45–C46

d C31–Al1–C45.

Despite the variable conditions of crystallization, the structures of all three alkenylalkynylaluminate products **1**–**3** display significant features in common and are isolated as molecular species in which the potassium cation is solvated either by two molecules of THF (**1** and **3**) or a molecule of η^6^-coordinated benzene (**2**). Like Roesky’s system (Scheme 1),^7,8^ the aluminum centers are four-coordinate and bound by C≡CR′ and PhCH=C­(R) groups, with proton addition to generate the vinylic substituent in both **2** and **3** having occurred at the less sterically encumbered Al–C­(Ph) bond of **VII**. In all three compounds the potassium cations interact similarly with the aluminate anions via a combination of η^2^- and η^6^-interactions with the alkynyl C≡C bond and the π system of a {SiN^Dipp^} ligand Dipp substituent.

### Reactivity toward CO_2_ and Ketones

Initial monitoring by ^13^C­{^1^H} NMR spectroscopy of benzene solutions of **VI** and **VII** with two atmospheres of CO_2_ provided comparable observations and the emergence of characteristic ^13^C labeled resonances at δ_C_ 175.4 (**VI**) and 176.5 ppm (**VII**). Although the reaction of **VI** reached completion after 3 days to provide a single new species (**4**), the similar generation of a *C*-silylated analog from **VII** was, despite the persistence of the signal at δ_C_ 176.5 ppm, complicated by the concurrent emergence of multiple additional species. The generation of an intractable mixture of compounds was most obviously manifested by two pairs of mutually coupled doublets at δ_C_ 169.5 and 186.7 ppm (*J* = 10.1 Hz) and 170.1 and 171.6 ppm (*J* = 9.3 ppm) (Figure S13). Although we can provide no definitive interpretation of this observation, we suggest the contrasting behavior of **VII** possibly results from an increased liability toward multiple CO_2_ insertion or reactivity induced by the oxophilicity of the trimethylsilyl substituent.

Removal of volatiles and crystallization of **4** from a mixture of hexane and THF provided crystals suitable for X-ray diffraction analysis. The structure depicted in Figure 3a, with selected bond length and angles in Table 2, revealed that each potassium is coordinated by a molecule of THF as part of a centrosymmetric dimer propagated by a sequence of K-κ^2^-carboxylate and K···Dipp π arene interactions. Although Roesky and co-workers’ study of [(BDI)­Al­{η^2^-C_2_(SiMe_3_)_2_}] with CO_2_ provided no NMR spectroscopic or X-ray analysis (Scheme 1),^6^ the constitution of **4** is otherwise analogous to their assigned structure, resulting from insertion of CO_2_ into one of the aluminacyclopropene Al–C bonds of **VI** (Scheme 3). The most notable feature of the carboxylatoaluminate structure is, thus, the newly formed five-membered heterocycle which features exocyclic C=O [C45–O2 1.2310(15) Å] and endocyclic C–O [C45–O2 1.2310(15) Å] bonds, and a C31–C32 distance [1.3525(17) Å] that reflects the maintenance of the C=C bond of **VI** in comparison to the newly formed C–C single bond [C32–C45 1.5005(15) Å].

**Figure 3:**
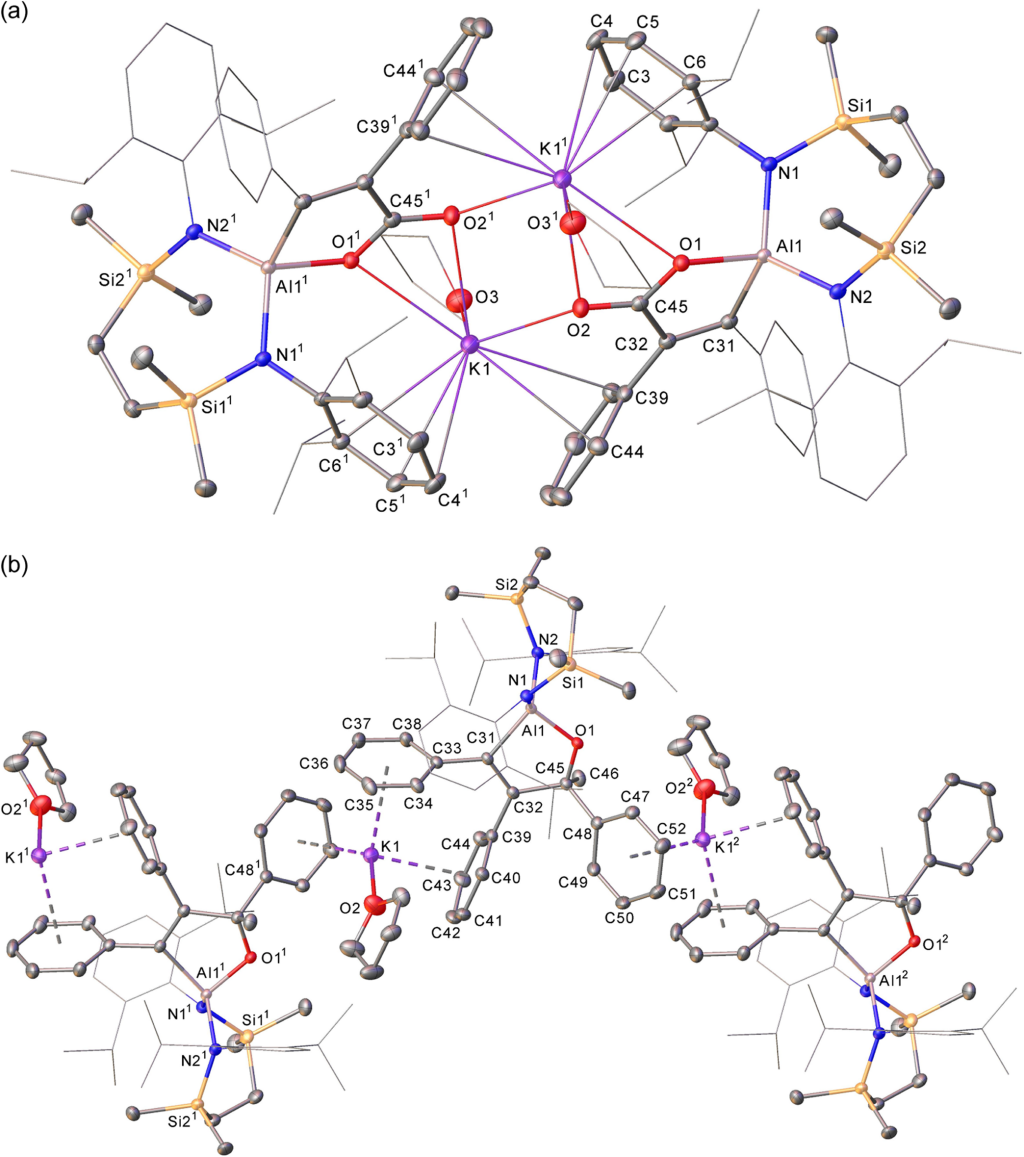
Displacement ellipsoid plots (30% probability) of (a) compound **4** and (b) a polymeric section of compound **6**. All hydrogens as well as minor component disordered atoms (**6**) have been removed for clarity. Wireframe view has been used for some groups in both compounds, also for visual ease. Symmetry operations to generate equivalent atoms (**4**) ^1^1 – *x*, 1 – *y*, 1 – *z*; (**6**) ^1^2 – *x*, −1/2 + *y*, 1 – *z*, ^2^2 – *x*, 1/2 + *y*, 1 – *z*.

**Scheme 3:**
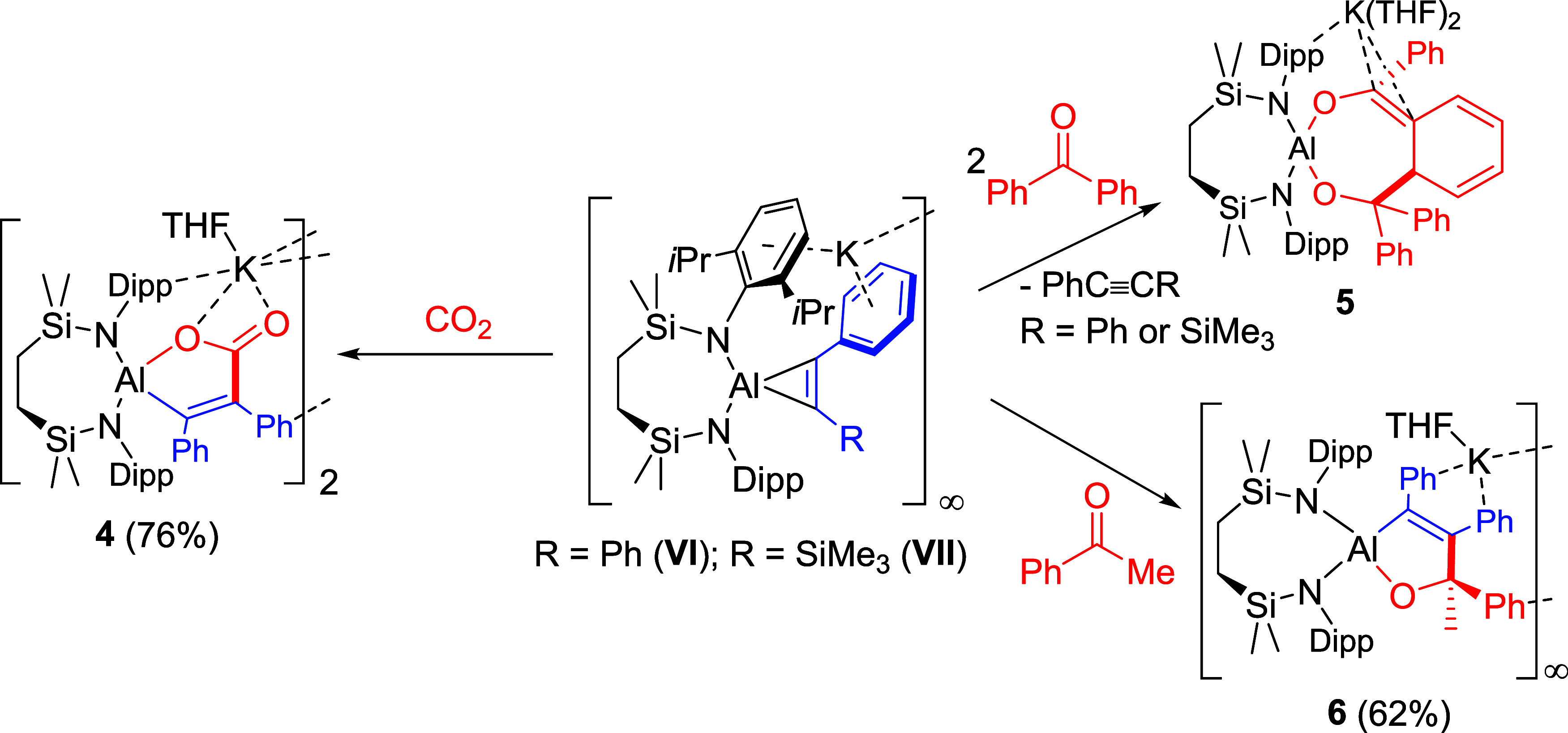
Synthesis of Compounds **4**–**6** (% Indicates Isolated Yield)

**Table 2:** Selected Bond Lengths (Å) and Angles (°) of Compounds **4**, **6**, **7**, **8**, **10**, and **11**

	**4**	**6**	**7**	**8**	**10**	**11**
Al1–N1	1.8550(10)	1.8750(19)	1.8712(14)	1.857(2)	1.8700(13)	1.862(2)
Al1–N2	1.8507(10)	1.8707(19)	1.8553(13)	1.853(2)	1.8575(13)	1.884(2)
Al1–C31	2.0188(12)	2.005(2)	1.9847(15)	2.011(3)	2.0010(15)	1.999(3)^h^
Al1–O1	1.8475(8)	1.7666(17)	1.9865(14)^b^	1.923(2)^b^	1.9358(13)^b^	1.966(2)^b^
C31–C32	1.3525(17)	1.347(3)	1.356(2)	1.346(4)	1.362(2)	1.354(4)
C32–C45	1.5005(15)	1.562(3)	1.4452(19)^c^	1.422(3)^c^	1.4208(19)^c^	1.421(3)^i^
C45–O1	1.3120(14)	1.395(3)	1.3367(17)^d^	1.308(3)^d^	1.3474(18)^d^	1.356(3)^d^
C45–O2	1.2310(15)	1.543(3)^a^	1.2710(18)^e^	1.309(3)^e^	1.299(2)^g^	1.290(4)^j^
N1–Al1–N2	113.46(4)	109.93(9)	112.60(6)	115.01(10)	112.47(6)	112.45(10)
C31–Al1–O1	86.82(4)	88.53(8)	69.62(6)^f^	69.48(11)^f^	70.21(6)^f^	70.81(10)^k^

a C45–C46.

b Al1–N3.

c C32–N3.

d N3–N4.

e N4–N5.

f C31–Al1–N3

g N4–C45.

h Al1–C32.

i C31–N3.

j N4–C42.

k C32–Al1–N3.

Monitoring of reactions of both **VI** and **VII** with a single equivalent of benzophenone by ^1^H NMR spectroscopy evidenced the apparent transformation of only *ca*. 50% of the aluminate starting materials alongside a new species (**5**), the reduced symmetry of which gave rise to a highly complex spectrum. In both cases, complete consumption of the aluminacyclopropene starting materials could be induced by use of two equivalents of the ketone reagent. Removal of the benzene reaction solvent and crystallization by slow evaporation of a THF solution allowed the isolation of single crystals of **5**, which was identified by a unit cell check as the known spirocyclic aluminate, K­(THF)_2_[(SiN^Dipp^)­Al-κ^2^-*O*,*O*’-{OCPh_2_CH­(CH =CHCH=CH)­C=CPhO}] (Scheme 3).^26^ Compound **5** was previously synthesized by treatment of the potassium alumanyl (**IV** ^**K**^, Figure 1) with benzophenone and, thus, may be considered as a product of reductive C–C coupling between a single ketyl carbon atom and an *ortho*-phenyl carbon of a second benzophenone moiety. Reminiscent of Roesky and co-workers’ reports of the reactivity of various [(BDI)­Al­{η^2^-C_2_(R^1^)­(R^2^)}] derivatives,^6^ therefore, this observation infers that, albeit by an unidentified mechanism, the aluminacyclopropene structures of **VI** and **VII** can behave as “masked” sources of the reductive Al­(I) center through the respective loss of diphenylacetylene or PhC≡CSiMe_3_.

In contrast to this latter deduction, reactions of **VII** with acetophenone provided no tractable products irrespective of the reaction stoichiometry. Treatment of **VI** with this ketone in benzene, however, induced the immediate precipitation of compound **6**, which was characterized by NMR spectroscopy in *d*_8_-THF and crystallized from a hexane/THF mixed solvent system. The resultant spectroscopic analysis provided data indicative of a low symmetry species, the structure of which was resolved by single crystal X-ray diffraction analysis (Figure 3b). Like **4**, compound **6** is a product of C=O insertion into a single Al–C bond of **VI**, but now with the concurrent generation of an asymmetric quaternary carbon center and resultant loss of mirror plane symmetry orthogonal to the [{SiN^Dipp^}­Al] heterocycle. The resultant spirocyclic alkoxyaluminate crystallizes as a one-dimensional polymer propagated by potassium ions that are coordinated by a molecule of THF and further encapsulated by three similar K···Ph interactions provided by the PhC=CPh unit of each aluminate and the formerly ketonic phenyl group of an adjacent anion. The composition of the five-membered Al-containing heterocycle is otherwise comparable to that of **5** with similar C–C [C32–C45 1.562(3) Å] and C=C bonds [C31–C32 1.347(3) Å]. Although we have no confident rationale for the contrasting formation of compounds **5** and **6**, these observations are consistent with a greater susceptibility of benzophenone to single electron reduction.

### Reactivity with *N*-Donor substrates

#### Organoazides

Reactions of compounds **VI** and **VII** were performed with adamantyl and mesityl azides, which were selected as representative alkyl- and arylazide reagents. Compound **VI** provided a similar outcome with both azides and the formation of azacyclobutenylaluminate derivatives, compounds **7** and **8**, by Al–C insertion of the terminal azide nitrogen (Scheme 4). This process of C–N bond formation is, therefore, analogous to Roesky’s reaction of [(BDI)­Al­{η^2^-C_2_H_2_}] with the bulky azide, 2,6-(2,4,6-*i*-Pr_3_–C_6_H_2_)_2_C_6_H_3_N_3_ (Scheme 1).^7^ The asymmetry introduced by the formation of these compounds, most clearly apparent in solution from the appearance of two discriminated (2H by relative integration) {SiN^Dipp^} *iso*-propyl methine septet resonances (δ 3.98 and 3.93 ppm) in the ^1^H NMR spectrum of **7**, was confirmed by X-ray diffraction analysis performed on single crystals isolated either from benzene (**7**) or hexane and THF (**8**) (Figure 4a,b).

**Figure 4:**
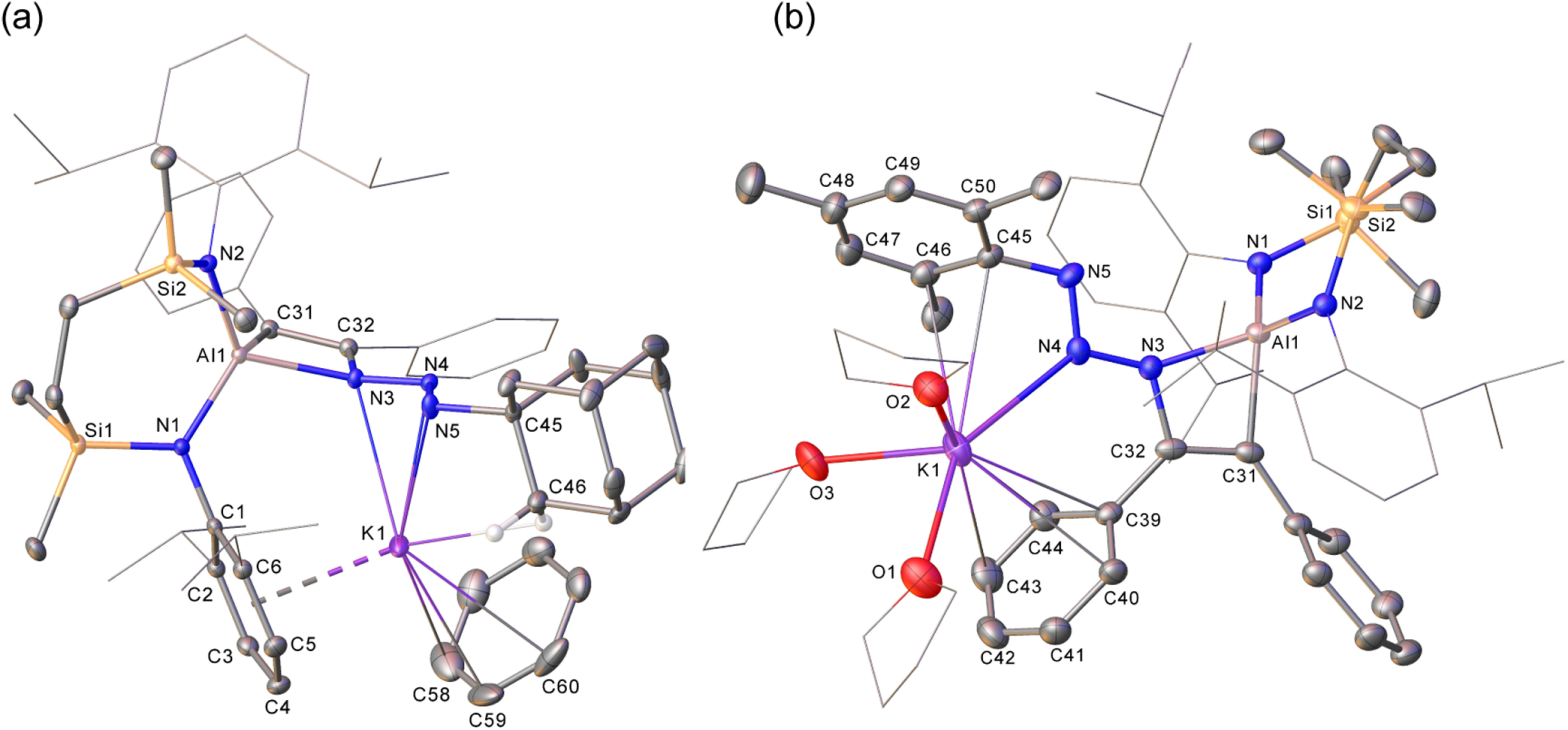
Displacement ellipsoid plots (30% probability) of (a) compound **7** and (b) compound **8**. The majority of hydrogens as well as minor component disordered atoms (**8**) and solvent (**7**) have been removed for clarity. Wireframe view has been used for some groups in both compounds, also for visual ease.

**Scheme 4:**
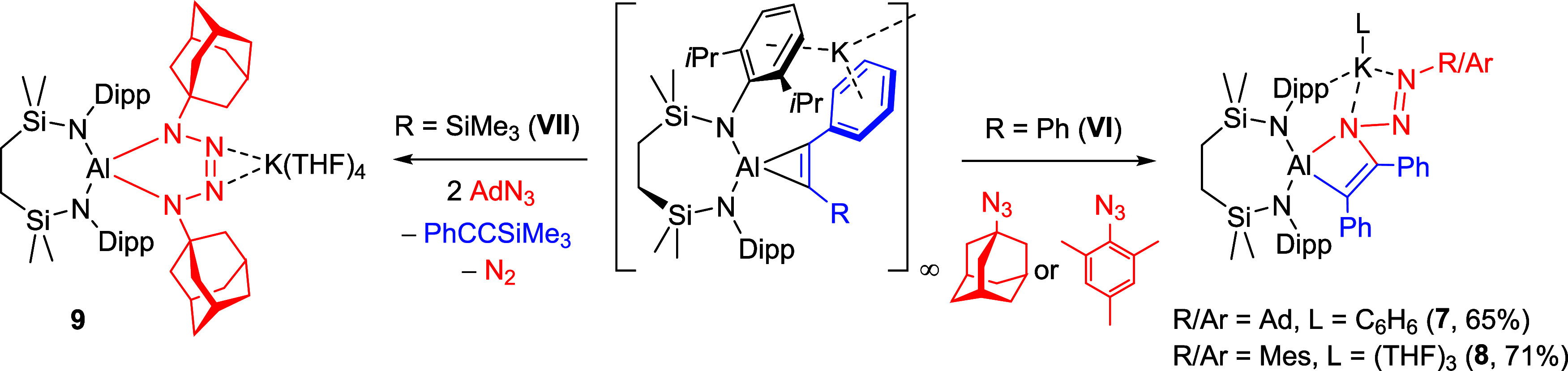
Synthesis of Compounds **7**–**9** (% Indicates Isolated Yield)

The structures of **7** and **8** comprise 4-membered rings formed by end-on azide insertion into an Al–C bond of the cyclopropene moiety. Although both compounds are similarly monomolecular, the K^+^ coordination sphere of **7** is completed by its η^6^-engagement of a molecule of benzene solvent, while that of **8** sequesters three molecules of THF. The potassium ion of **7** interacts with a flanking *N*-Dipp group of the {SiN^Dipp^} ligand and displays a η^3^-*N*_3_ triaza-allyl-like engagement [K1–N3 2.8668(14), K1–N4 2.8088(13), K1–N5 2.9519(16) Å] with the N3, N4 and N5 nitrogen atoms [N3–N4 1.3367(17), N4–N5 1.2710(18) Å]. The resultant main feature of difference between the two structures is, thus, provided by the contrasting κ^1^ interaction between N4 and the potassium cation of **8**, which resides in an environment further defined by the *N*-Mes and C-Ph π systems. While this also results in an apparently higher degree of delocalization across the former azido N_3_ unit [N3–N4 1.308(3), N4–N5 1.309(3) Å] of **8**, both derivatives again display C31–C32 bond lengths [1.356(2) Å (**7**), 1.346(4) Å (**8**)] indicative of the maintenance of the aluminacyclopropenyl double bond of **VI**.

In contrast to this behavior, reactions of the more sterically encumbered **VII**, provided a more complex outcome. Addition of MesN_3_ provided evidence of an intractable range of products irrespective of reaction stoichiometry. Reaction of **VII** with two equivalents of AdN_3_, however, resulted in expulsion of PhC≡CSiMe_3_ and, after analysis by NMR spectroscopy in *d*_8_-THF, the identification of the tetrazolylaluminate species **9** (Scheme 4). We have previously reported that compound **9** may be synthesized by reaction of the azide with the potassium alumanyl (**IV** ^**K**^).^27^ Compound **9**, therefore, may be considered as a further demonstration of the ability of **VII** to behave as a latent source of reducing Al­(I) character by extrusion of, in this instance, the more sterically encumbered trimethylsilylacetylene.

#### Trimethylsilyldiazomethane

The groups of Coles and Kinjo have demonstrated that the treatment of both the potassium derivative of **III** (Figure 1) and a related cyclic (alkyl)­(amino)­alumanyl anion with diazoalkanes can induce N_2_ elimination and the respective generation of unusual ketamide and alumina-alkylidene species.^28,29^ Accordingly, benzene solutions of both derivatives were treated at room temperature with a hexane solution of trimethylsilyldiazomethane (Scheme 5).

**Scheme 5:**
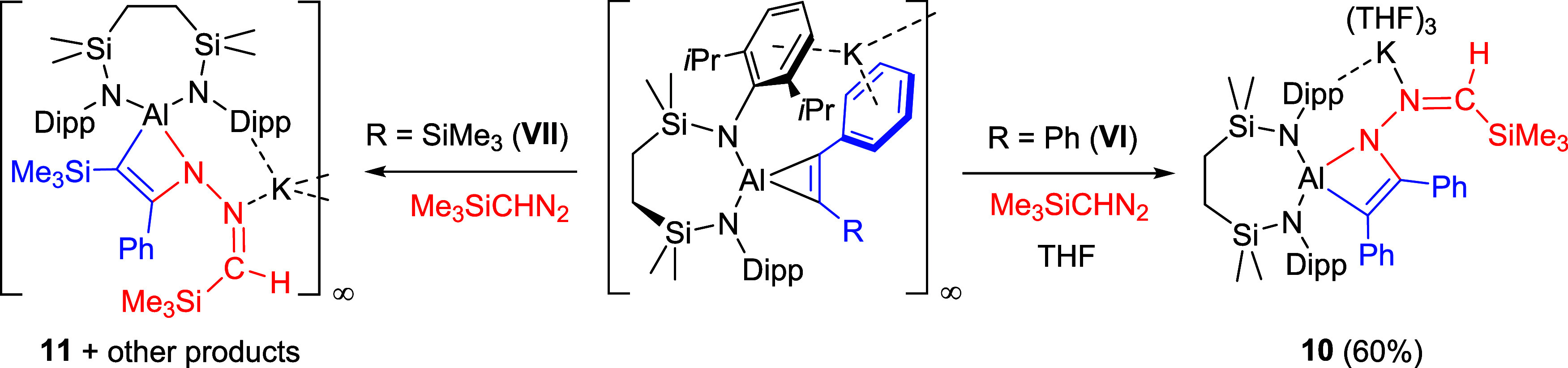
Synthetic Routes to Compounds **10** and **11** (% Indicates Isolated Yield)

Although neither system evidenced any visible gas evolution, the reaction with **VI** deposited a colorless precipitate of compound **10** over the course of 24 h. This material was subsequently insoluble in arene solvents but dissolved readily in *d*_8_-THF. In a similar manner to the spectra provided by compound **7**, the observation of two *N*-Dipp methine septet resonances at δ 4.08 and 3.95 ppm in the ^1^H NMR spectrum were consistent with loss of *C*_*2*_ symmetry about the [{SIN^Dipp^}­Al] moiety. The diasterotopic silylmethyl environments of the {SiN^Dipp^} manifested as two singlets at δ 0.12 and 0.01 ppm, both of which resonated in a 6:6:9 ratio of intensities relative to a further upfield singlet signal at −0.05 ppm. These observations, in conjunction with the appearance of a (1H) singlet at δ 5.89 ppm, assigned as a vinylic methine proton, were thus indicative of the continued integrity of the {(Me_3_Si)­HC} unit of the diazoalkane starting material. This deduction was corroborated by X-ray diffraction analysis performed on a single crystal of **10** grown by slow diffusion of hexane into a THF solution.

The result of this analysis (Figure 5a) confirmed that compound **10** is a monomeric ion paired compound in which three equivalents of THF are coordinated to each potassium. Charge balance is maintained by an azacyclobutenylaluminate anion formed by insertion of the terminal nitrogen of the diazomethane into one of the Al–C bonds of **VI**. The N3–N4 [1.3474(18) Å] and N4–C45 [1.299(2) Å] bond lengths are more consistent with significantly localized single and double bonds, respectively. Although aluminum-centered reactivity of this type does not appear to have been observed previously, while by no means common, examples of similar 1,1-additions have been reported for a variety of group 4 ^30–32^ and 4f-^33^ and 5f-centered^34–36^ organometallics.

**Figure 5:**
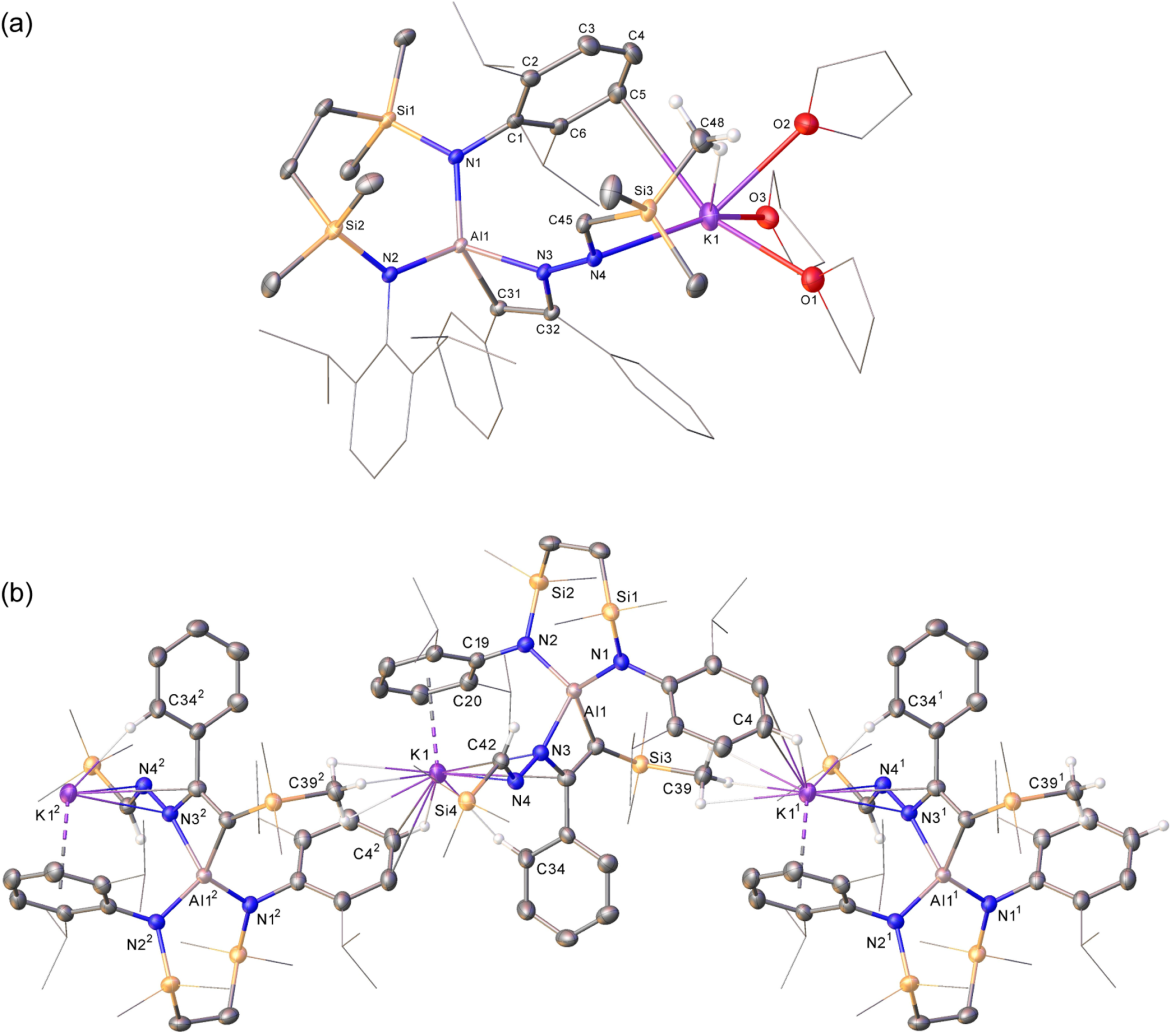
Displacement ellipsoid plots (30% probability) of (a) compound **10** and (b) the polymeric structure of compound **11**. Hydrogen atoms (except those of C48 and C45 of **10** and C4, C34, C39 and C42 of **11**) and disordered atoms have been removed for clarity. Wireframe view has been used for some groups on both compounds for visual ease. The majority of hydrogens as well as minor component disordered atoms have been removed for clarity. Wireframe view has been used for some groups in both compounds, also for visual ease. Symmetry operations to generate equivalent atoms (**11**) ^1^1 - *x*, −1/2 + *y*, 1/2 – *z*, ^2^1 – *x*, 1/2 + *y*, 1/2 – *z*.

Although assessment of the reaction of **VII** with Me_3_SiCHN_2_ by ^1^H NMR spectroscopy indicated the formation of a complex mixture of products (Figure S24), the benzene reaction solution remained homogeneous leading to the deposition of several crystals of compound **11**. Although insufficient in quantity for further assessment by NMR spectroscopy, this material was suitable for an X-ray diffraction analysis that revealed its identity as [{{SiN^Dipp^}­Al-κ^2^-C,N-(C­(SiMe_3_)=C­(Ph)-N­(NCHSiMe_3_))}­K]_∞_ (Figure 5b). Like **10**, compound **11** is a product of insertion of the terminal diazomethane nitrogen, in this case at the less sterically encumbered Al–C­(Ph) bond of **VII**. Although this THF-free variant crystallizes as a one-dimensional polymer propagated by *N-*Dipp···bridging between adjacent aluminate moieties, the similarity of the structure of **11** with that of **10** obviates further necessary comment. This structure, however, is unrepresentative of the entirety of the reaction, possibly due to competitive diazomethane insertion at the silyl-substituted Al–C bond alongside potential loss of PhC≡CSiMe_3_ and diazomethane reduction in a similar manner to that inferred from the isolation of compound **9**.

## Conclusions

The potassium cyclopropenylaluminates, [{SiN^Dipp^}­Al-η^2^-(C_2_Ph_2_)­K] and [{SiN^Dipp^}­Al-η^2^-(PhC=CSiMe_3_)­K], react with terminal alkynes to provide alkynylvinylaluminate derivatives. While the silyl-substituted variant provides a level of kinetic discrimination that results in retention of the more sterically congested Al–C­(SiMe_3_) bond, reactions with CO_2_ and phenyl-substituted ketones are complicated by a tendency toward multiple C=O insertion or loss of coordinated alkyne. This latter process results in reactivity more reminiscent of the Al­(I) compounds used to synthesize the AlC_2_ heterocycles. Similar observations arise from reactions with organic azides and trimethylsilyldiazomethane, which proceed with selective terminal nitrogen insertion for [{SiN^Dipp^}­Al-η^2^-(C_2_Ph_2_)­K] but with evidence of competitive alkyne elimination from [{SiN^Dipp^}­Al-η^2^-(PhC=CSiMe_3_)­K]. While we have no specific rationale for this dissonant reactivity, we note that the greater inclination toward “masked” Al­(I) behavior prevails for the more encumbered system, prompting the tentative conclusion that the process of alkyne reductive elimination is, at least in part, sterically induced. We are continuing to study these and related aluminate derivatives.

## Supplementary Material



## Notes

NMR spectra and crystallographic data (PDF)
