# Supplementary material for: Reactivity of Cyclopropenylaluminates
Source: Organometallics. 2025 Aug 27;44(17):1987–97. doi: 10.1021/acs.organomet.5c00272 (PMC12421673; doi:10.1021/acs.organomet.5c00272)
Supplement: Supplementary file 1 [file om5c00272_si_001.pdf]

Supporting Information for

Reactivity of Cyclopropenylaluminates

Marco F. Starostzik, Jakub Kenar, Han-Ying Liu, Mary F. Mahon and Michael S. Hill*

Department of Chemistry, University of Bath, Claverton Down, Bath, BA2 7AY, United Kingdom

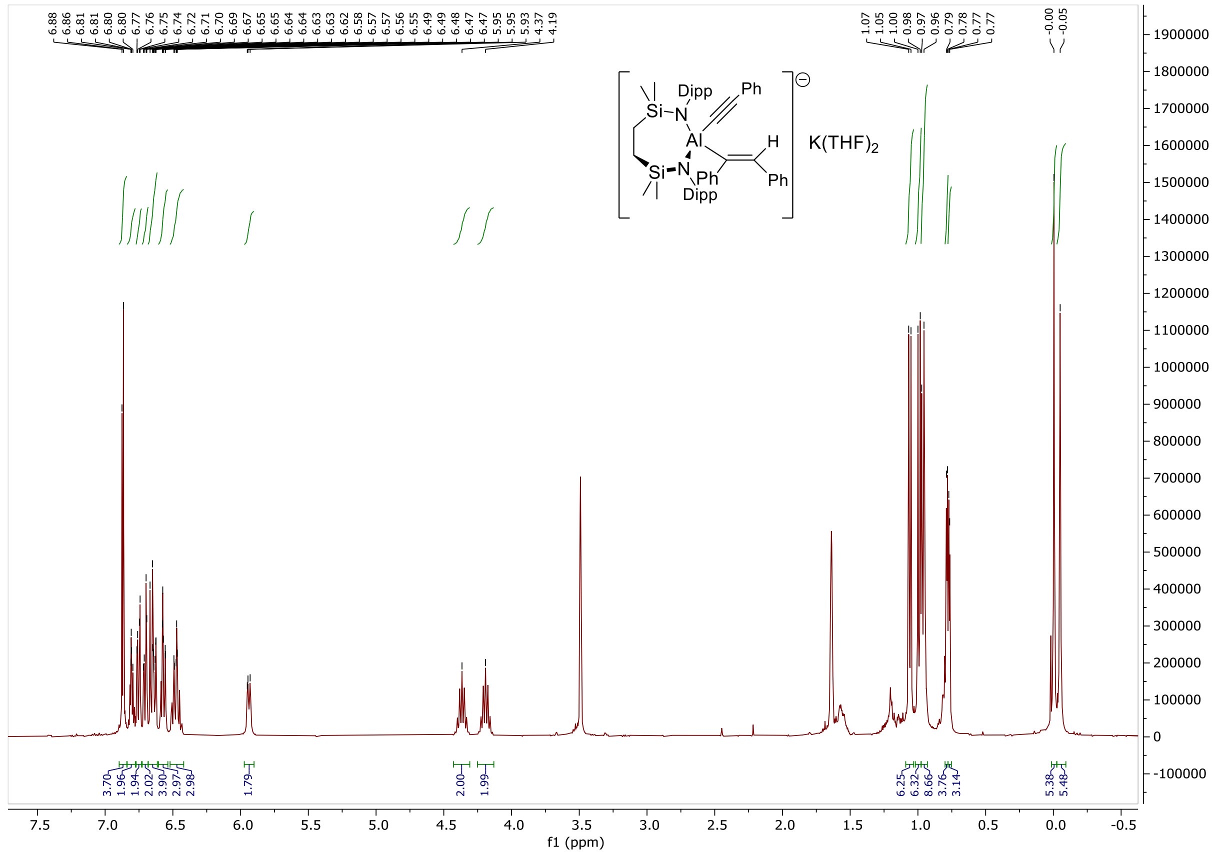

Figure S1. ^{1}H NMR (400 MHz, 298 K, d_8-THF) spectrum of **1**.

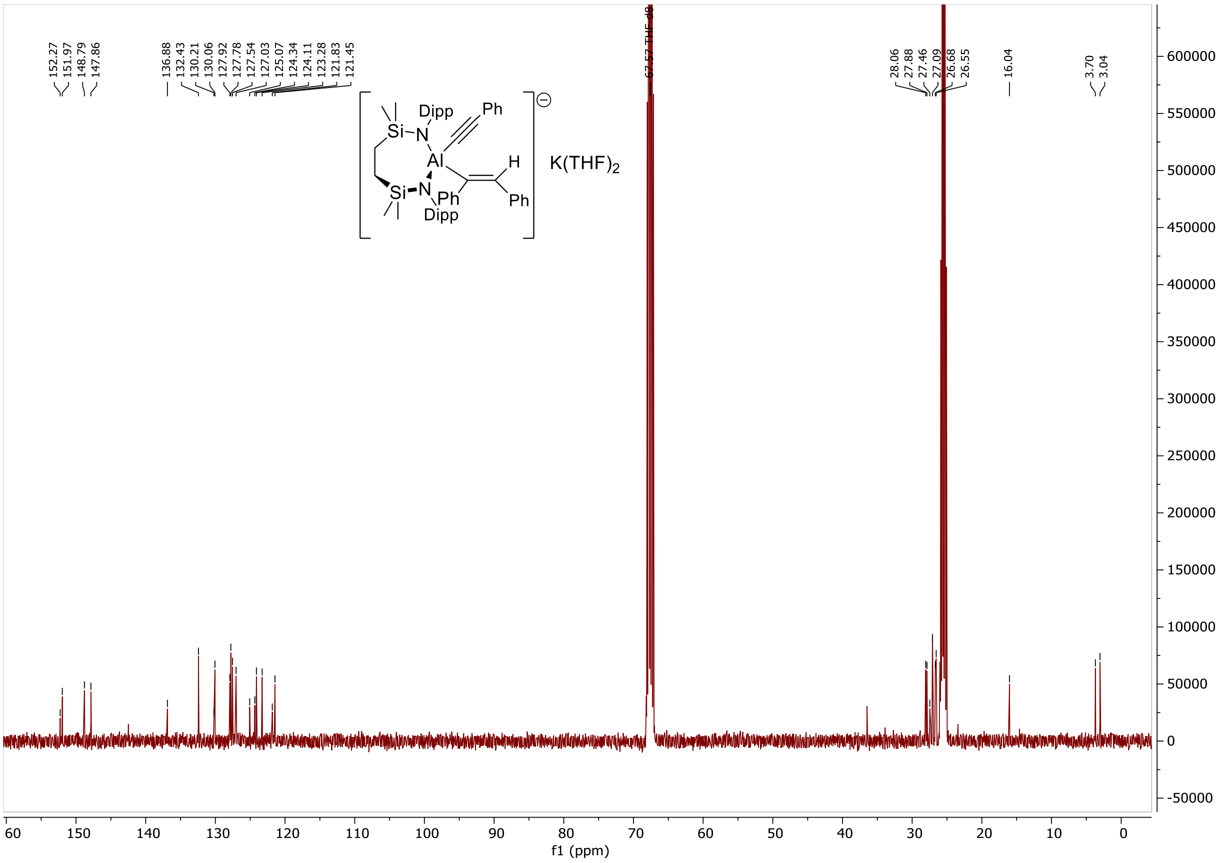

Figure S2. ^{13}C{^{1}H NMR (101 MHz, 298 K, d_8-THF) spectrum of **1**.

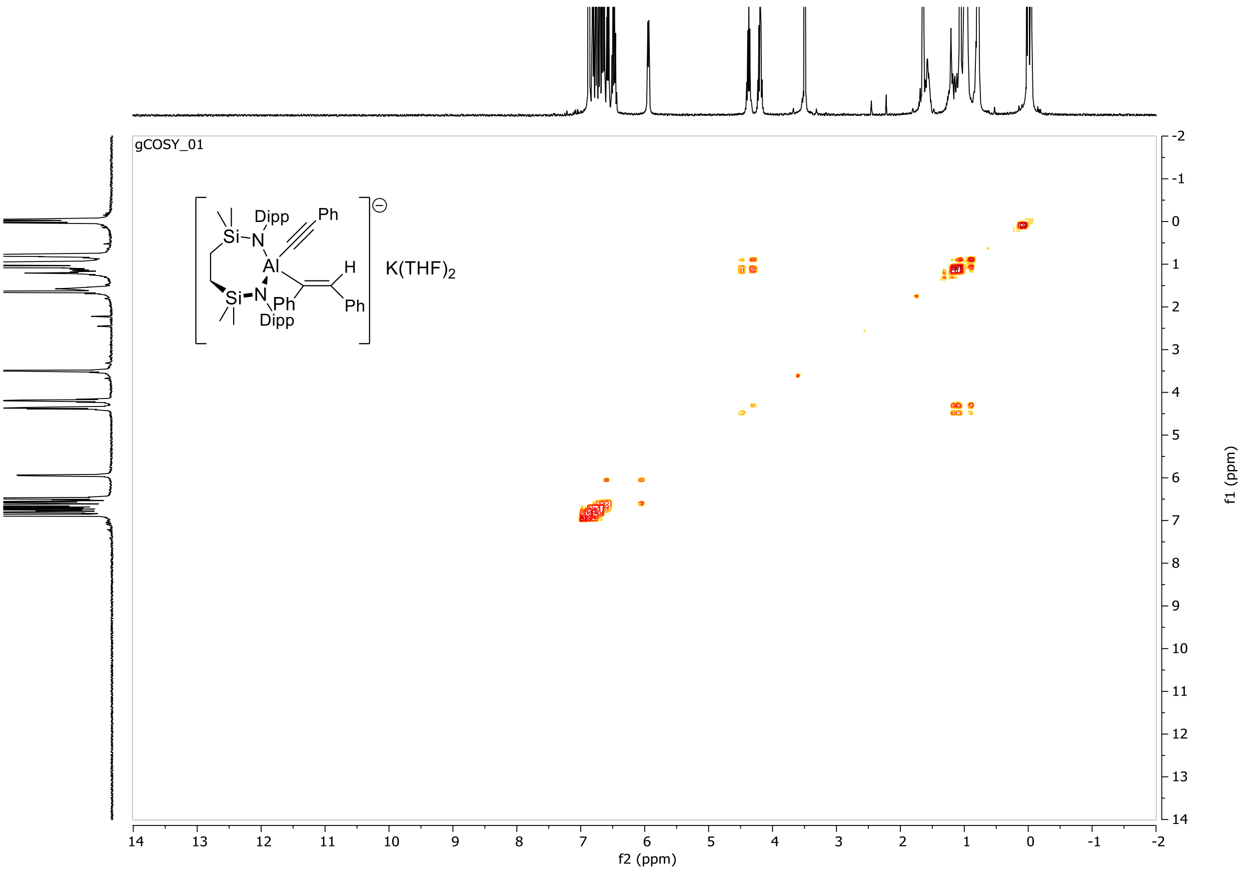

Figure S3. ^{1}H-^{1}H NMR COSY (298 K, *d*$_8$-THF) trace of **1**.

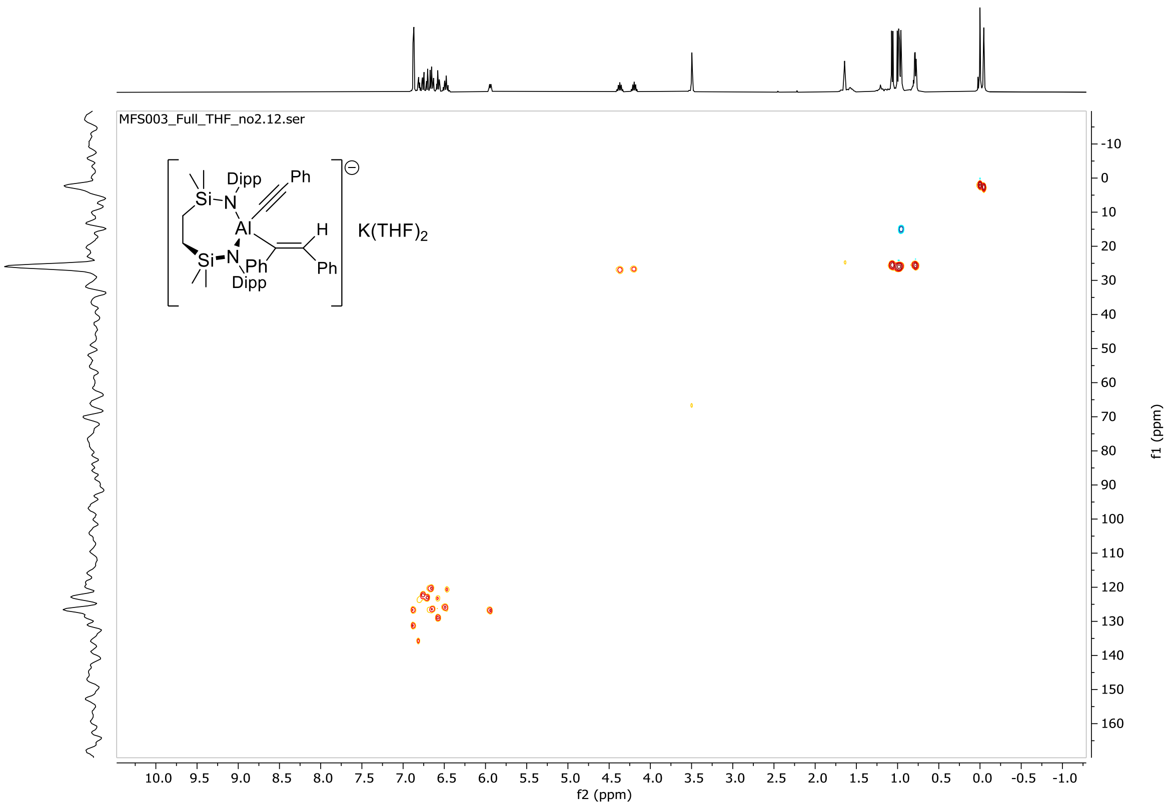

Figure S4. ^{1}H-^{13}C NMR HSQC (298 K, *d*$_8$-THF) trace of **1**.

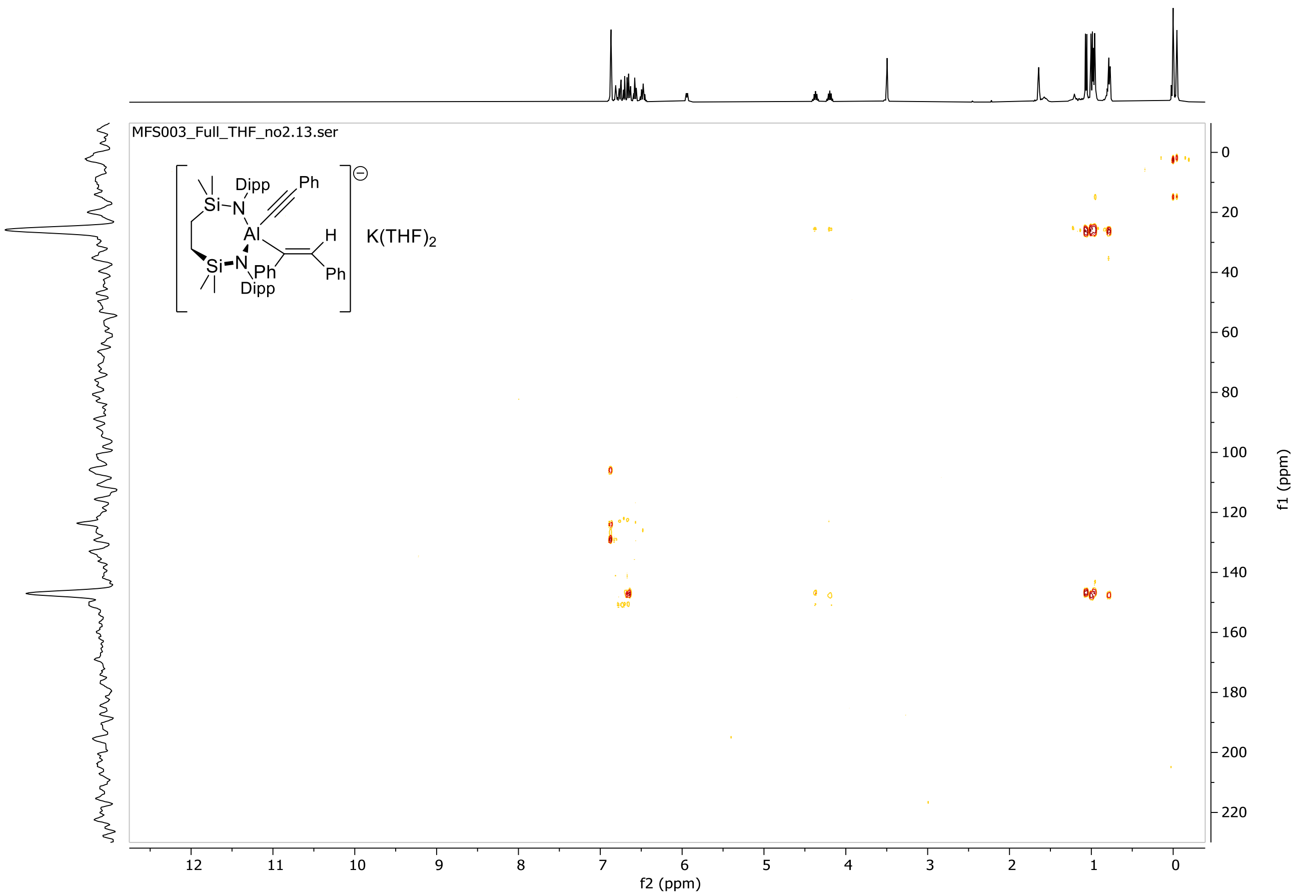

Figure S5. ^{1}H-^{13}C NMR HMBC (298 K, d_8-THF) trace of **1**.

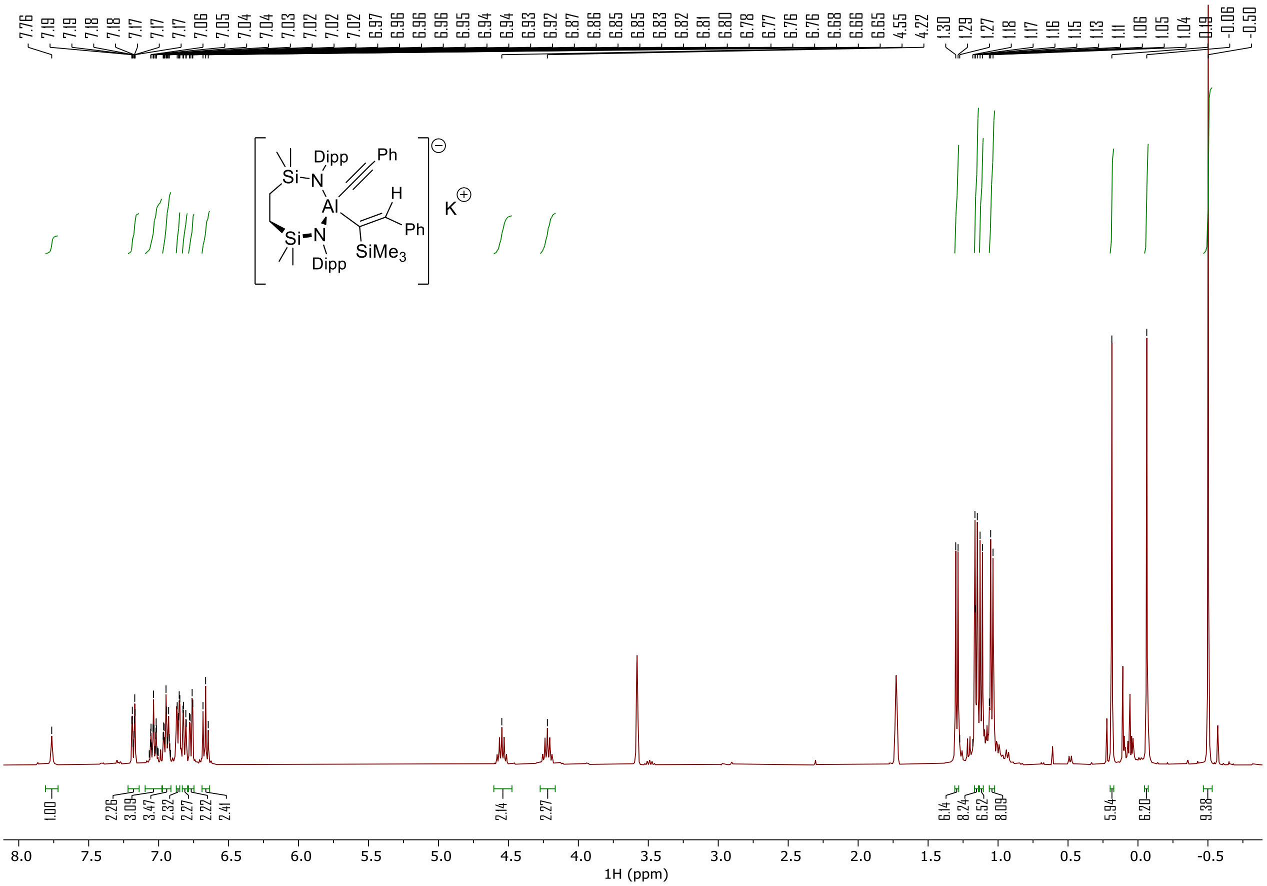

Figure S6. ^{1}H NMR (400 MHz, 298 K, d_8-THF) spectrum of **2**.

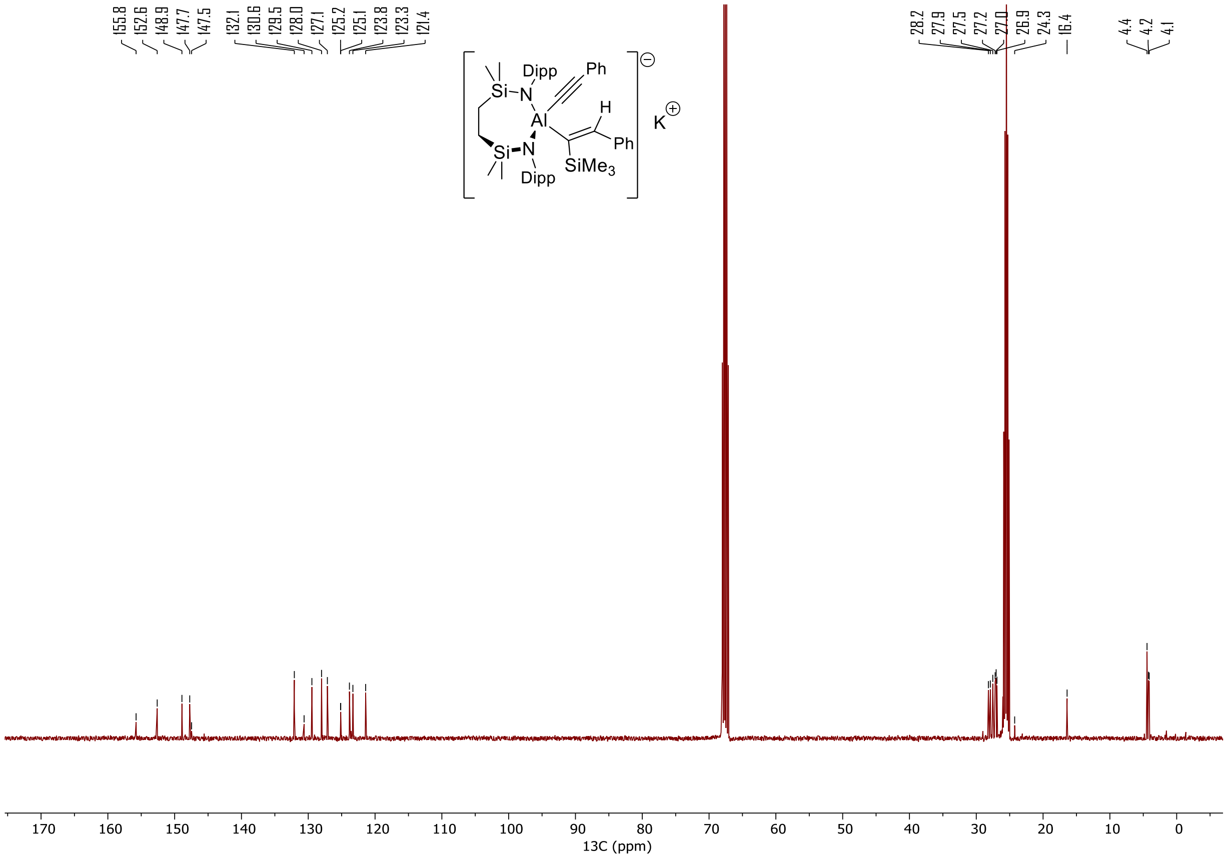

Figure S7. ^{13}C{^{1}H} NMR (101 MHz, 298 K, d_8-THF) spectrum of **2**.

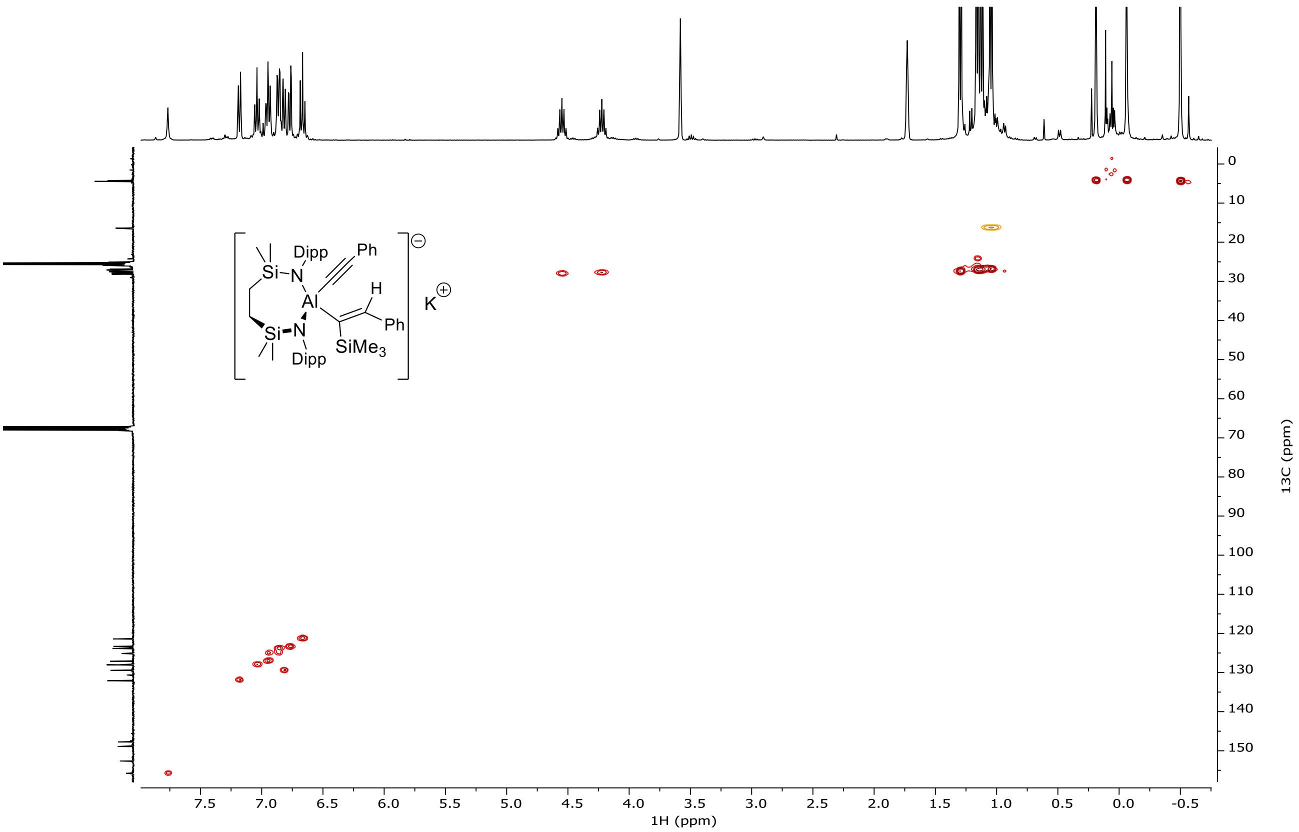

Figure S8. ^{1}H-^{13}C NMR HSQC (298 K, d_8-THF) trace of **2**.

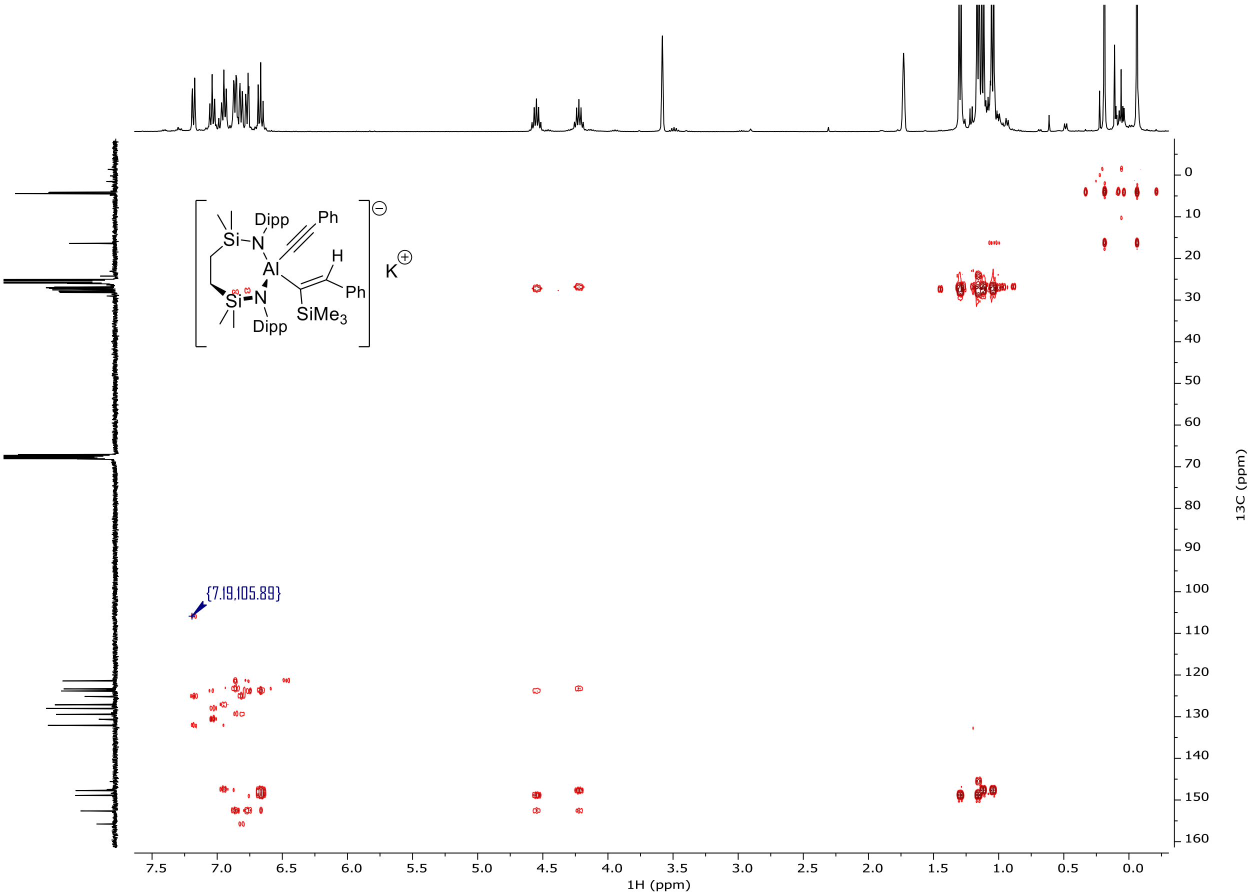

Figure S9. ^{1}H-^{13}C NMR HMBC (298 K, d_8-THF) trace of **2**.

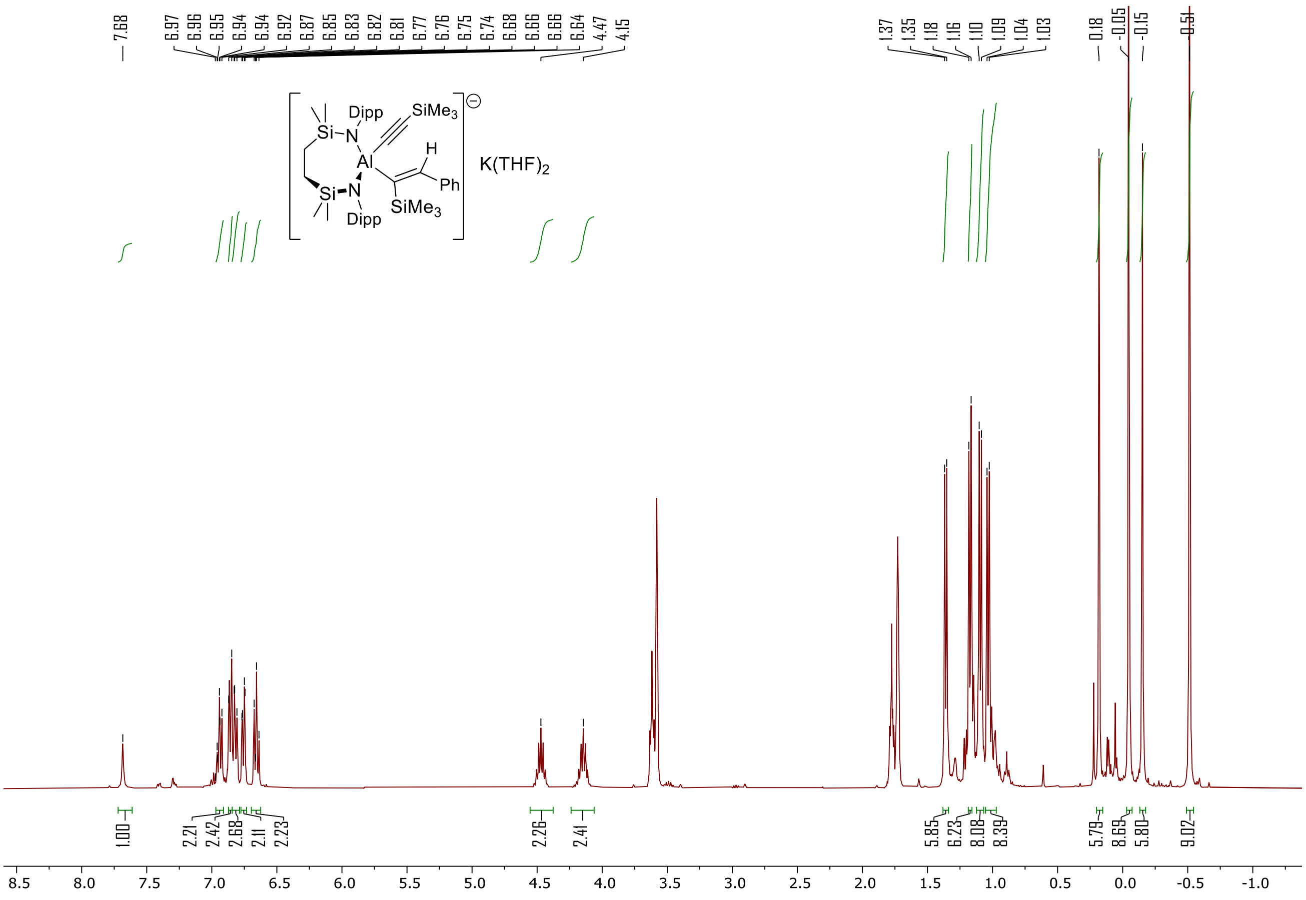

Figure S10. ^{1}H NMR (400 MHz, 298 K, d_8-THF) spectrum of **3**.

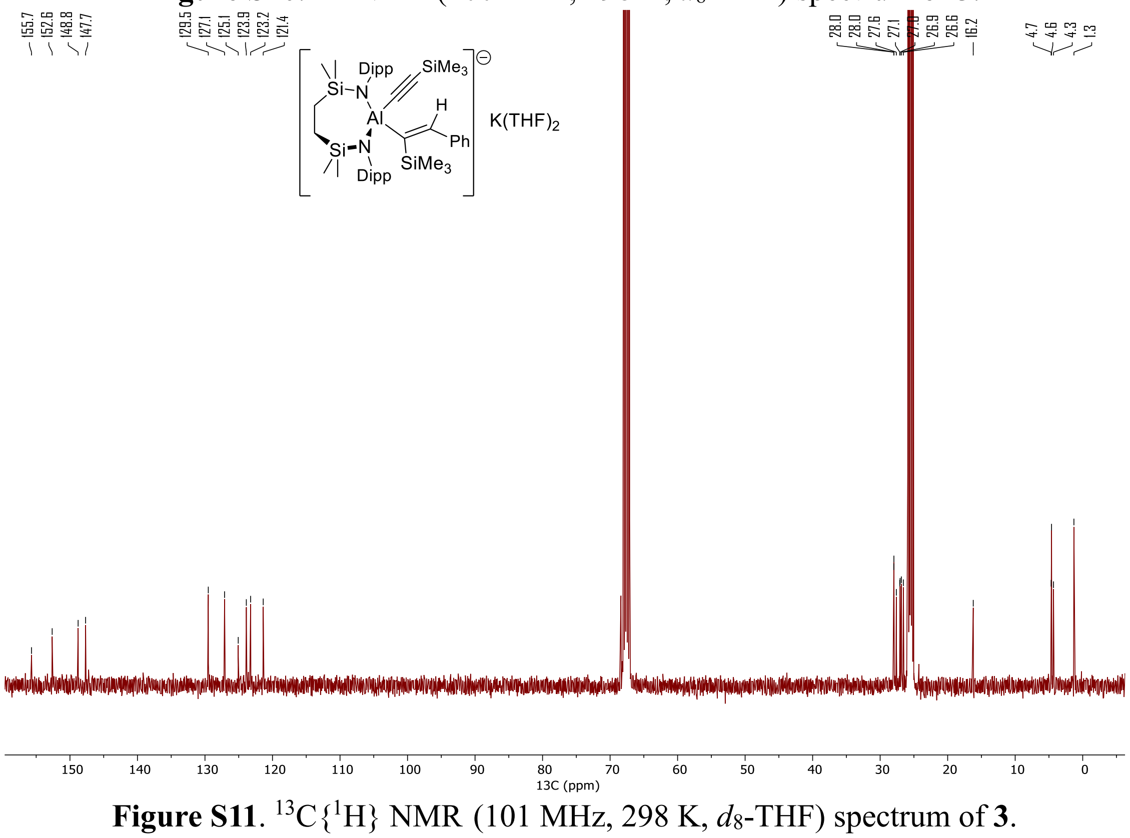

Figure S11. ^{13}C{^{1}H} NMR (101 MHz, 298 K, d_8-THF) spectrum of **3**.

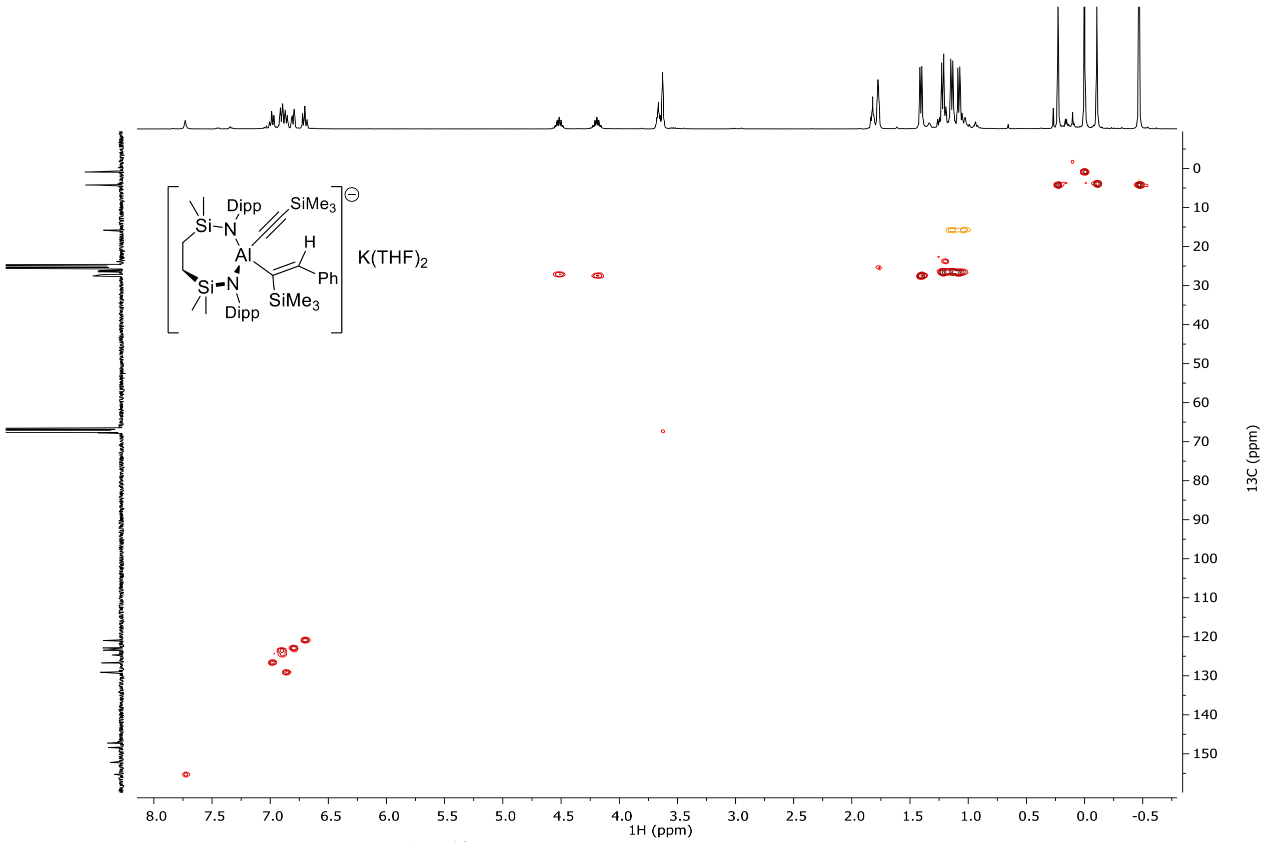

Figure S12. ^{1}H-^{13}C NMR HSQC (298 K, d_8-THF) trace of **3**.

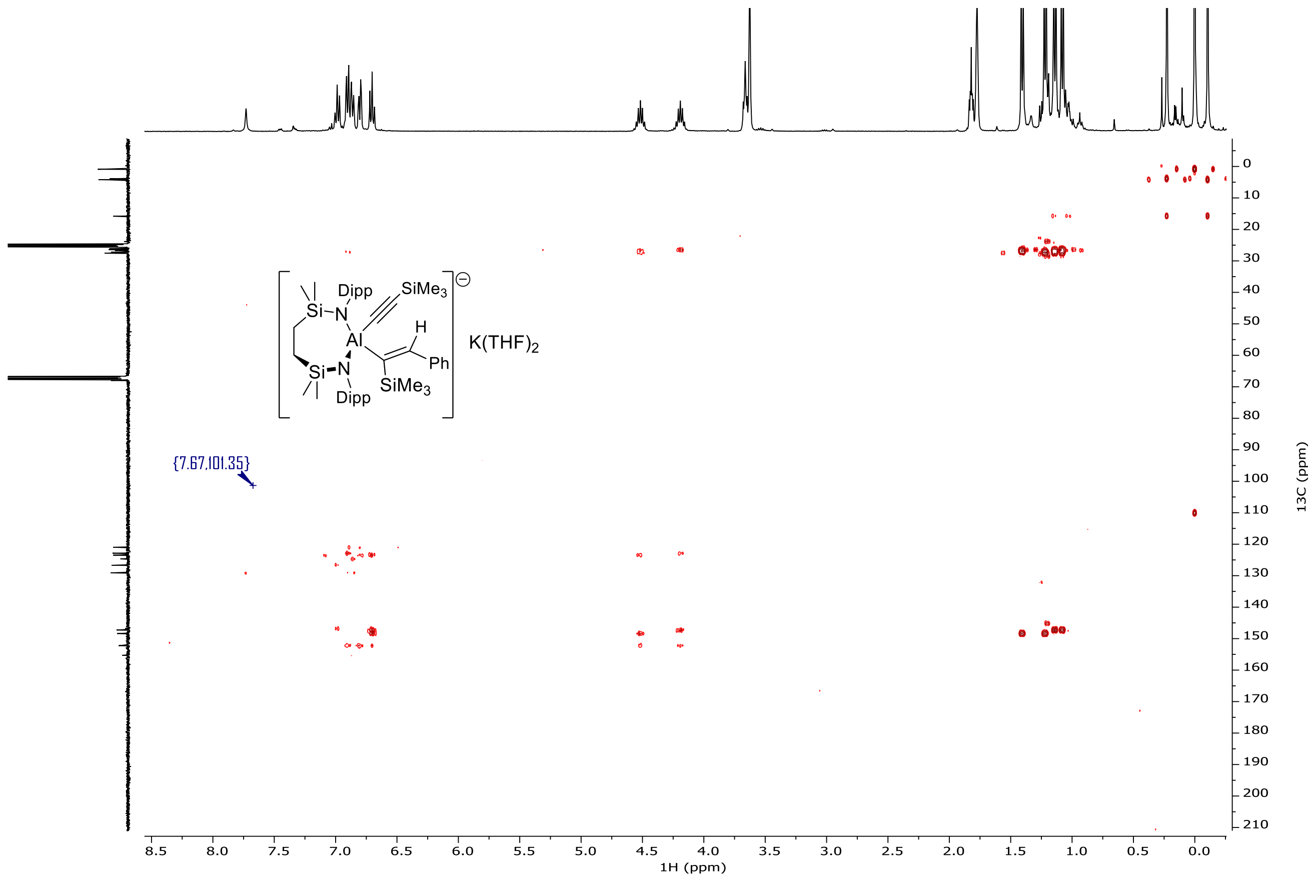

Figure S13. ^{1}H-^{13}C NMR HMBC (298 K, d_8-THF) trace of **3**.

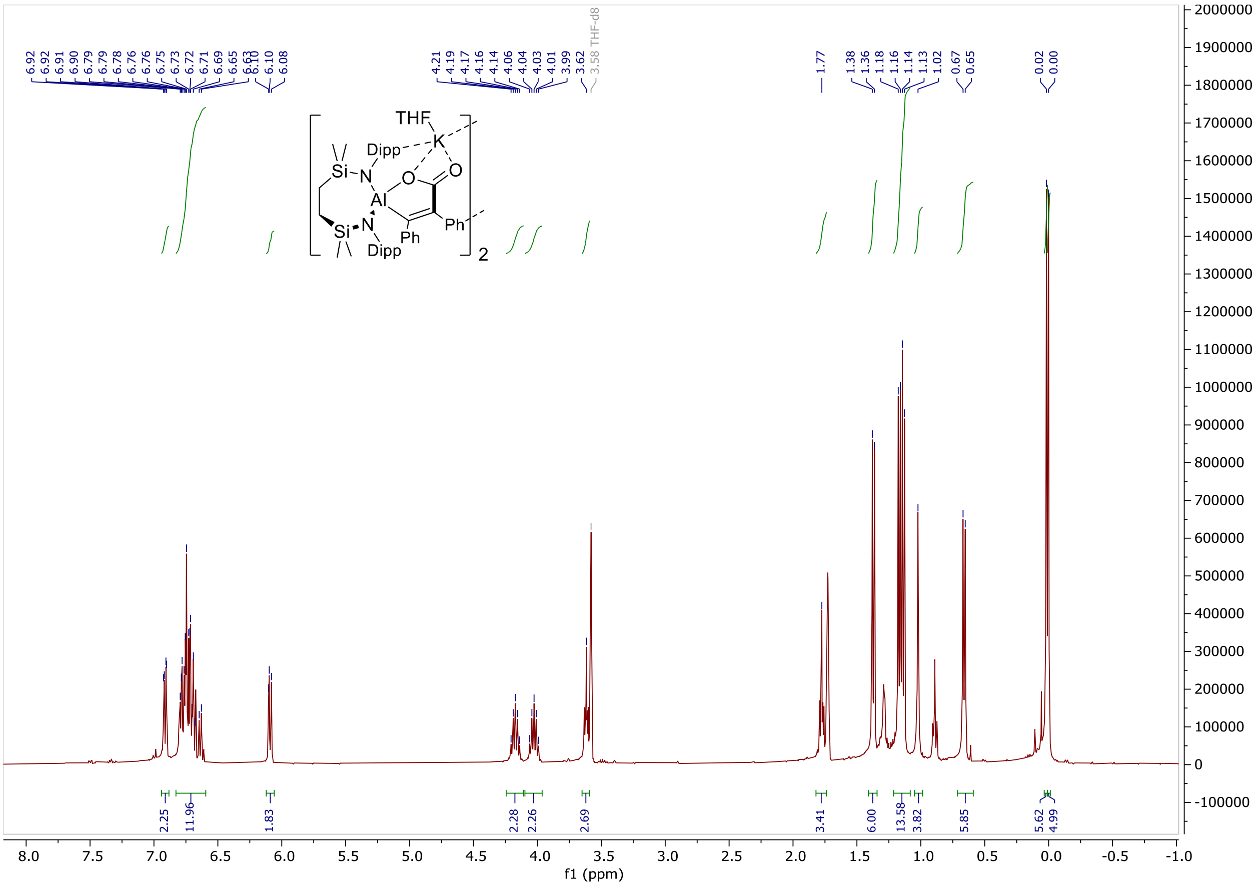

Figure S14. ^{1}H NMR (400 MHz, 298 K, d_8-THF) spectrum of **4**.

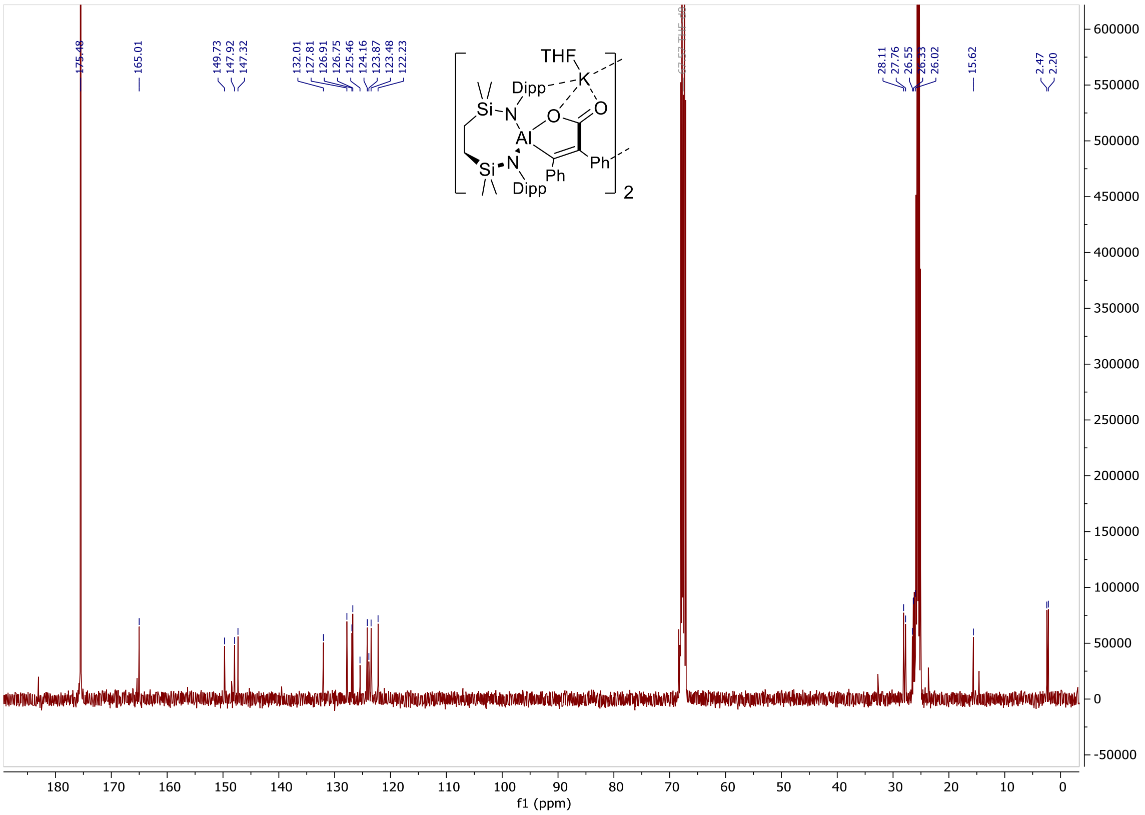

Figure S15. ^{13}C{^{1}H} NMR (101 MHz, 298 K, d_8-THF) spectrum of **4**.

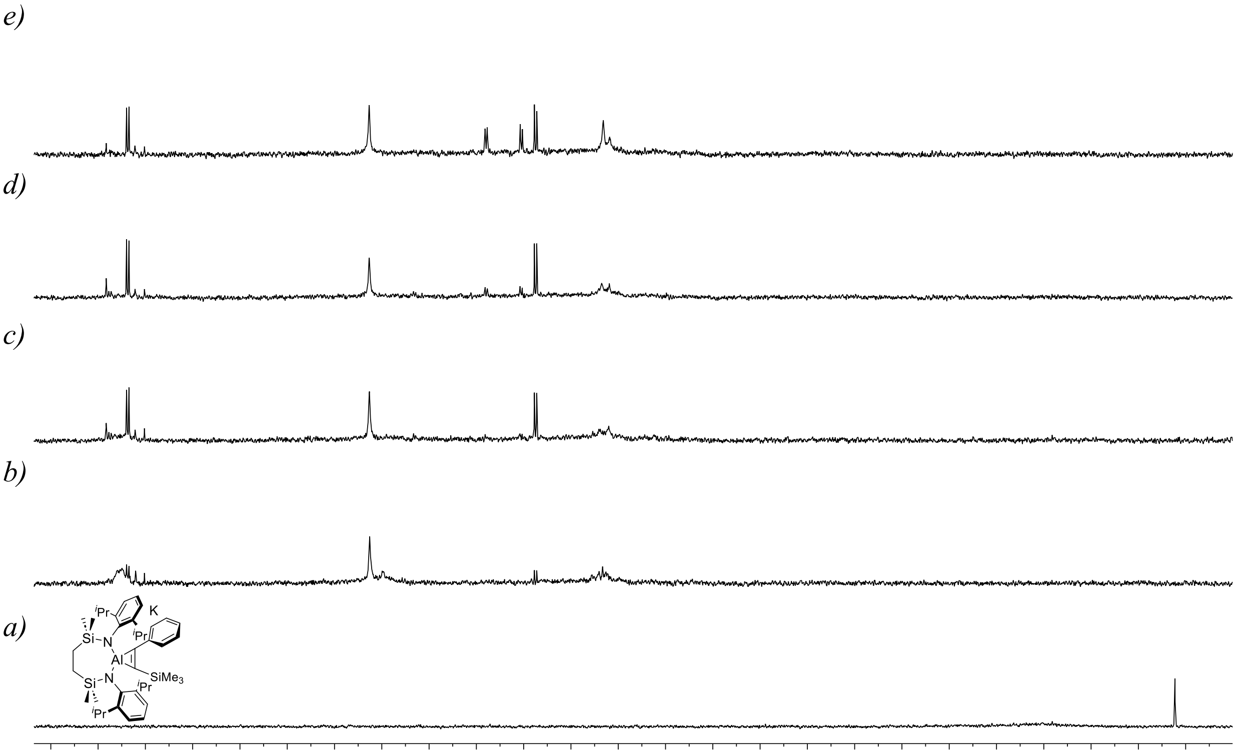

Figure S16. $^{13}C\{^{1}H\}$ NMR spectra (101 MHz, 298 K, d_8-THF) of the reaction between [{SiN^{Dipp}}Al-η^2-*C*,*C'*-(PhCCSiMe$_3$)K] (**VII**) (*a*) and CO_2 after *b*) initial reading; *c*) 1 day; *d*) 2 days; *e*) 2 weeks.

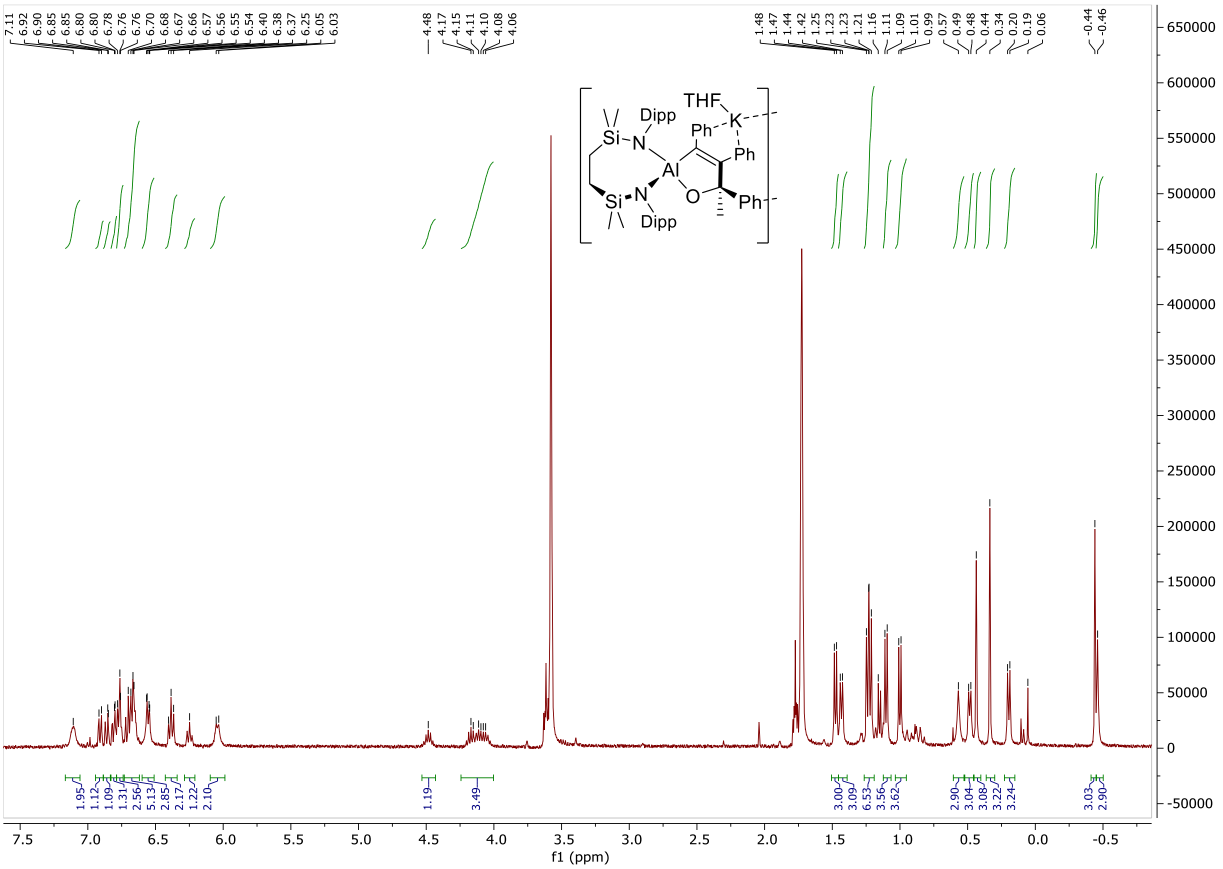

Figure S17. ^{1}H NMR (400 MHz, 298 K, d_8-THF) spectrum of **6**.

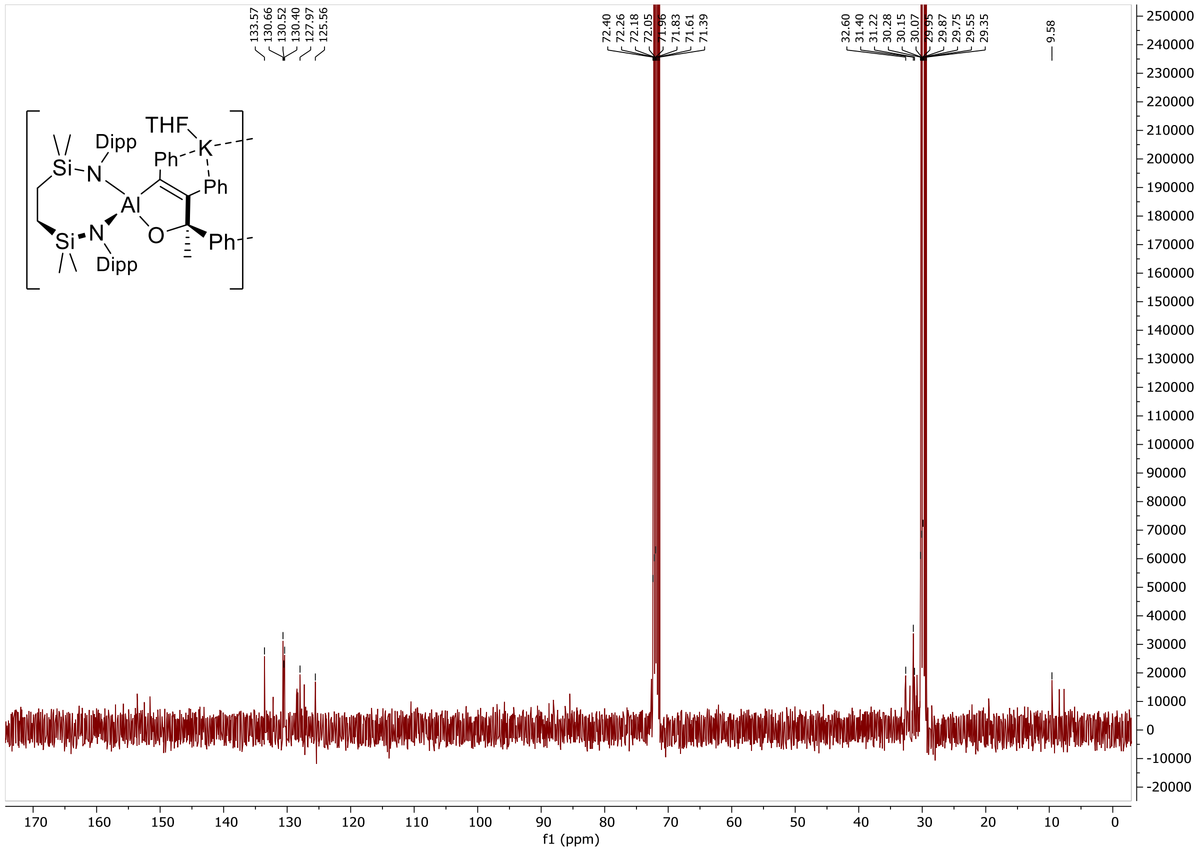

Figure S18. ^{13}C{^{1}H} NMR (101 MHz, 298 K, d_8-THF) spectrum of **6**.

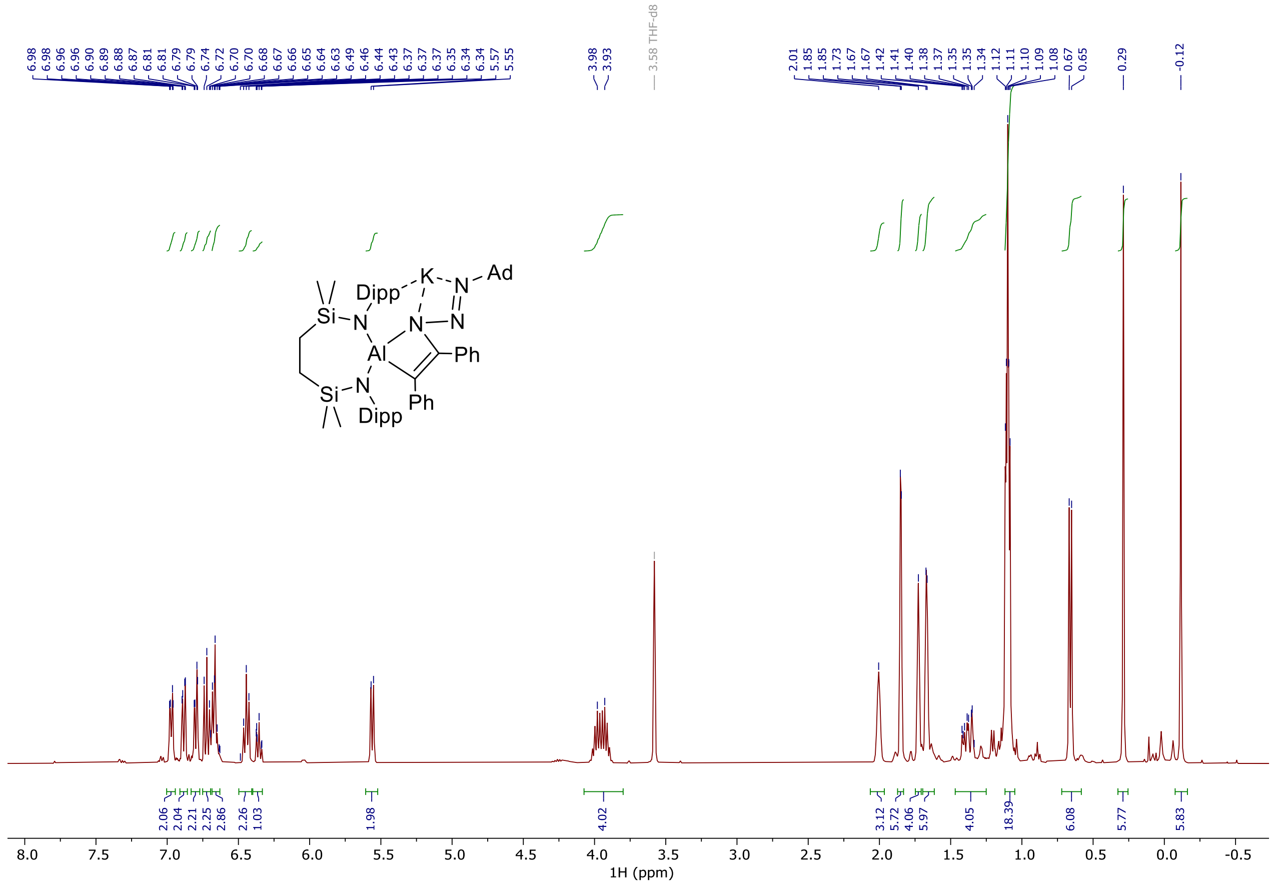

Figure S19. ^{1}H NMR (400 MHz, 298 K, d_8-THF) spectrum of **7**.

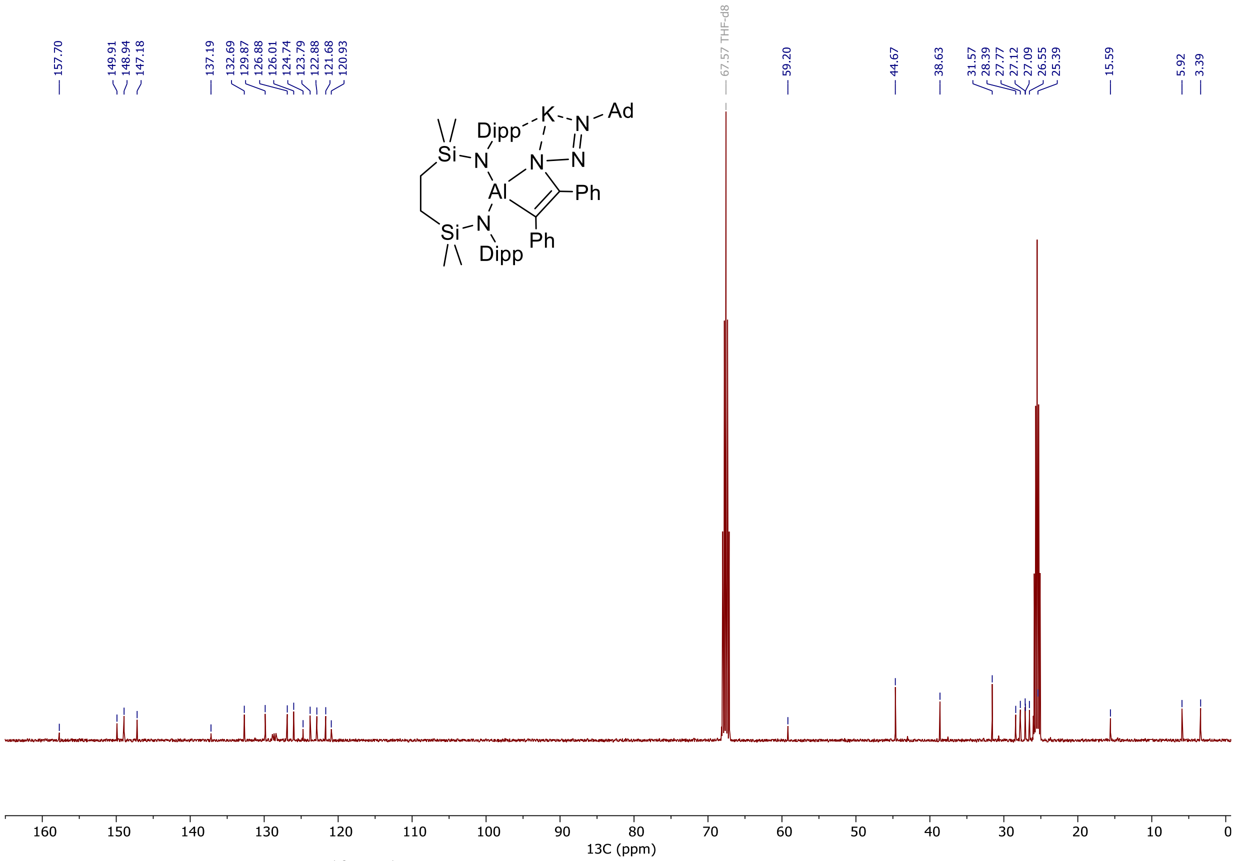

Figure S20. $^{13}C\{^{1}H\}$ NMR (101 MHz, 298 K, d_8-THF) spectrum of **7**.

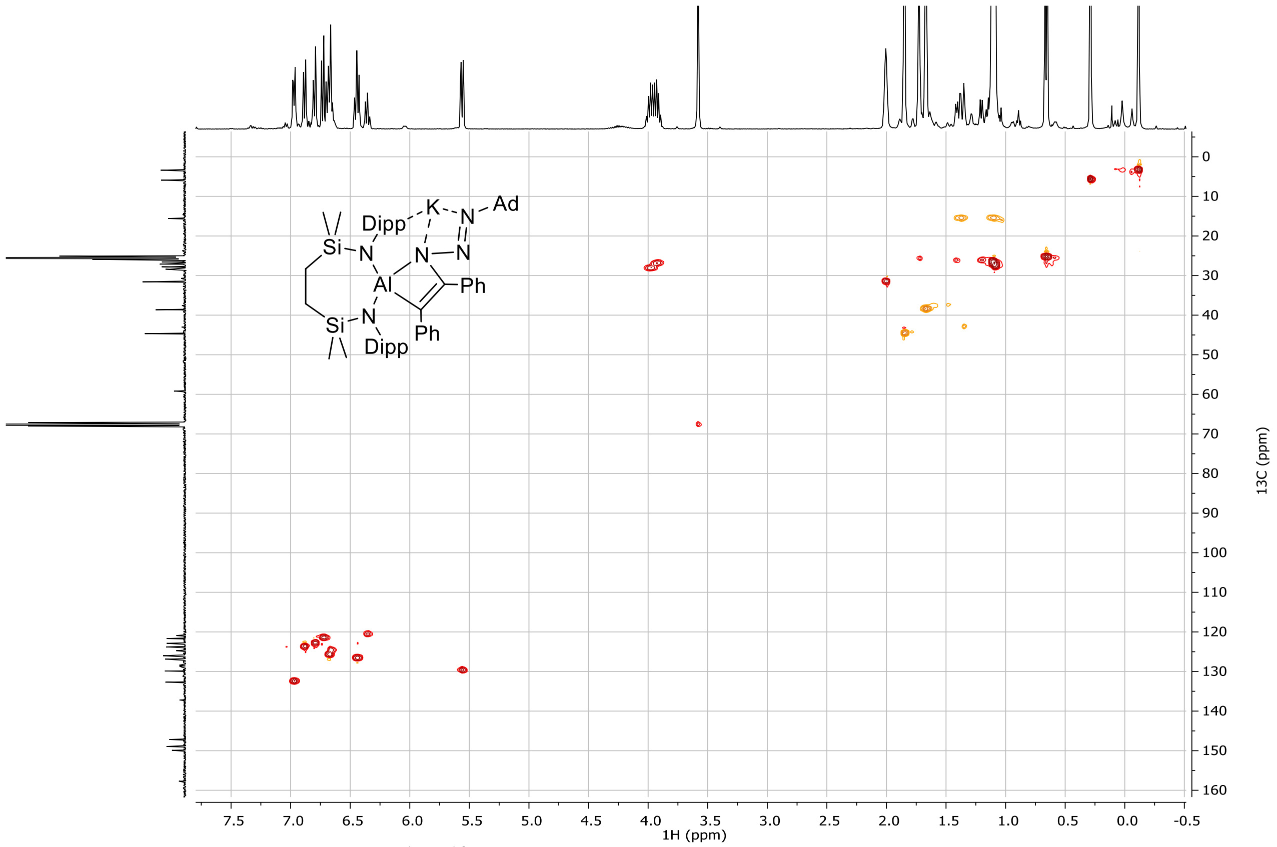

Figure S21. ^{1}H-^{13}C NMR HSQC (298 K, d_8-THF) traces of **7**.

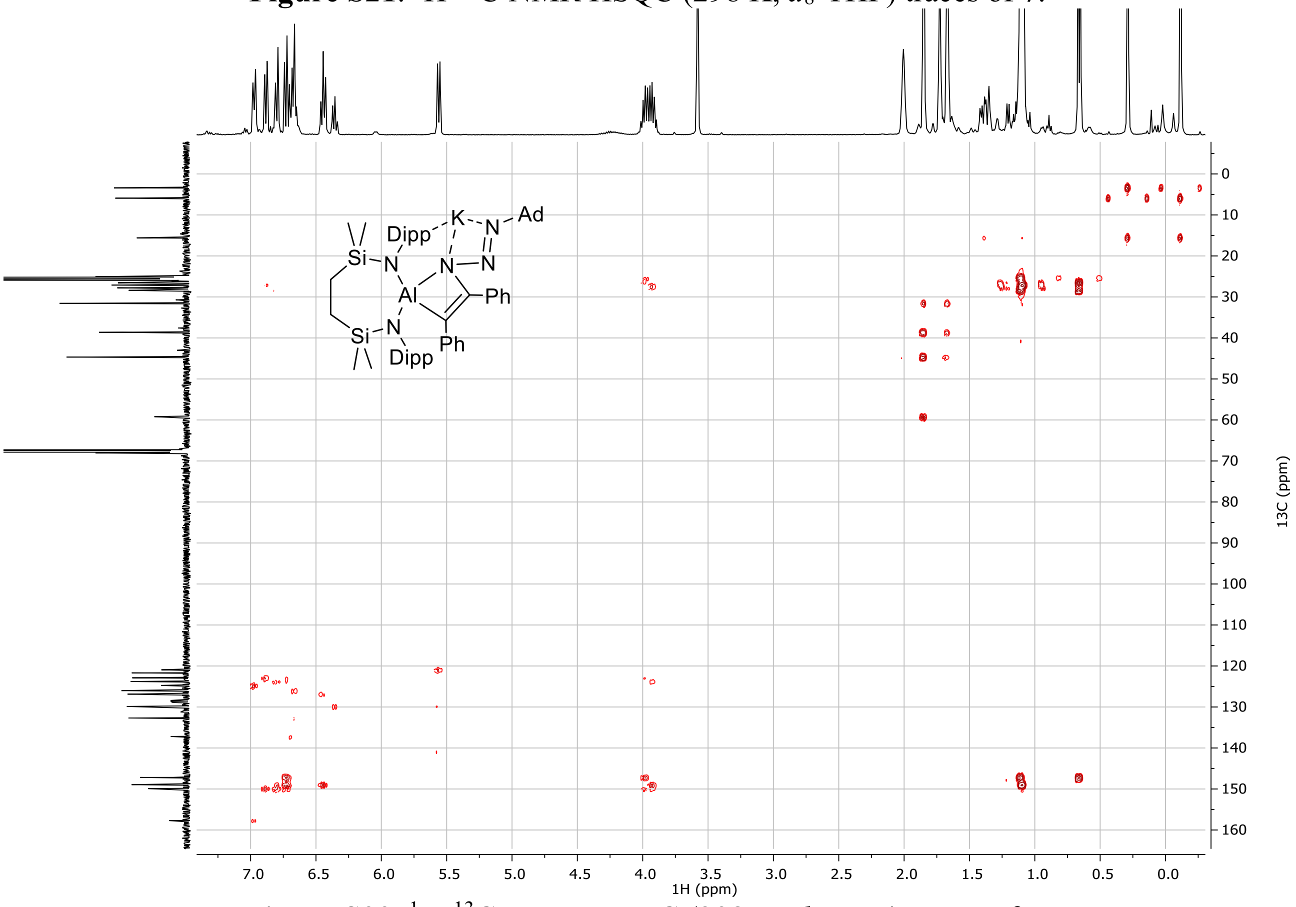

Figure S22. ^{1}H-^{13}C NMR HMBC (298 K, d_8-THF) traces of **7**.

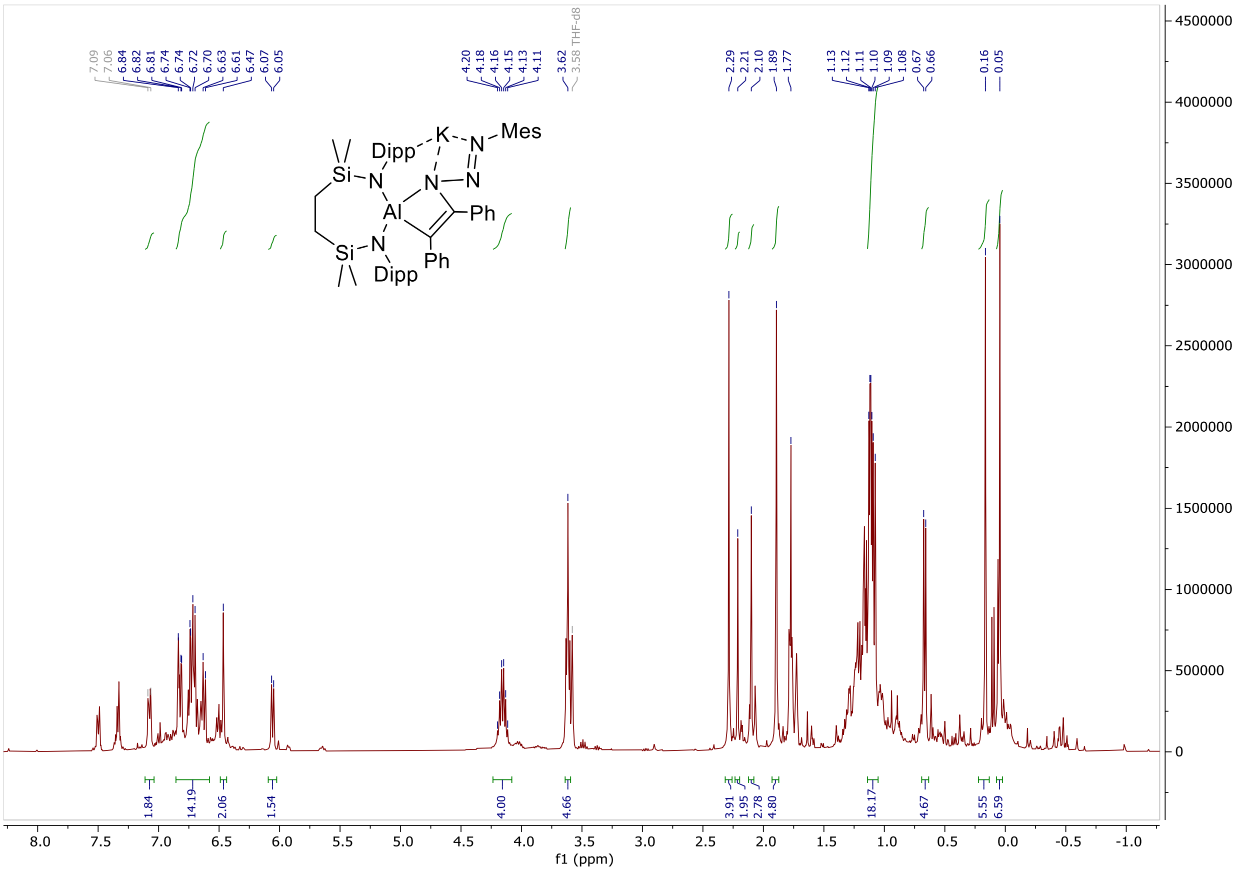

Figure S23. ^{1}H NMR (400 MHz, 298 K, d_8-THF) spectrum of **8**.

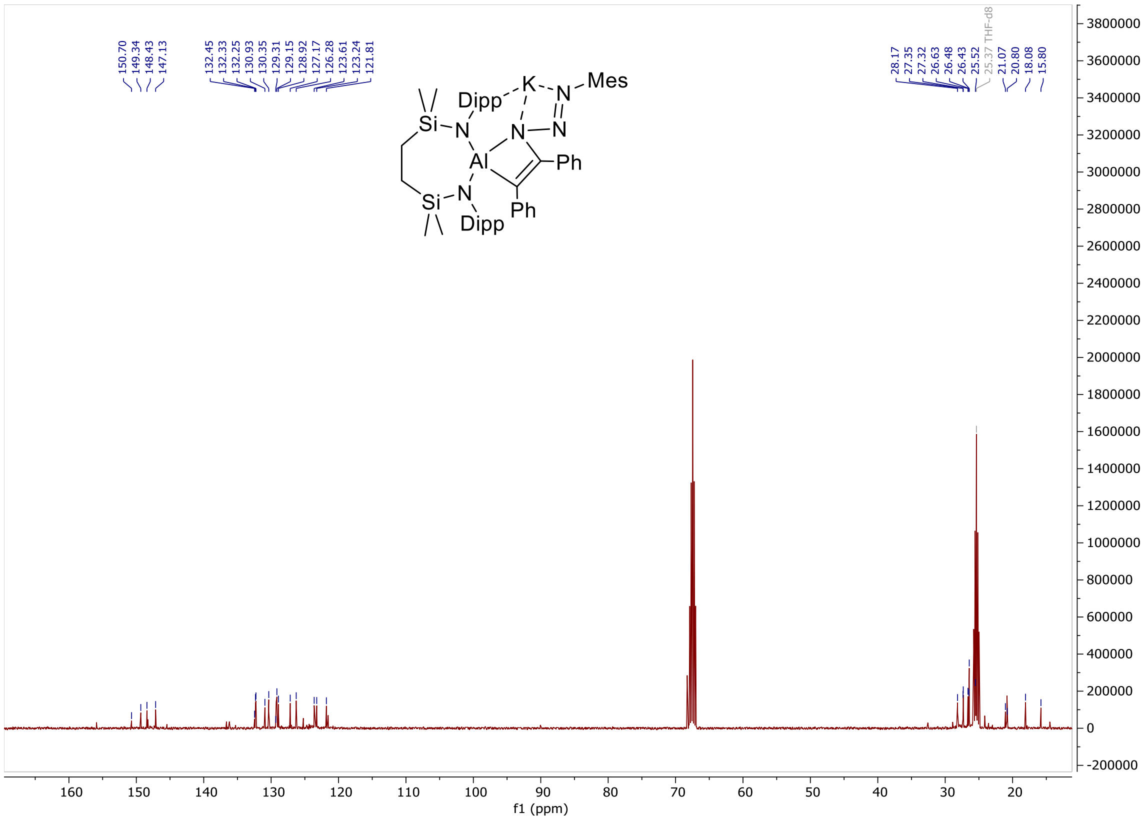

Figure S24. $^{13}C\{^1H\}$ NMR (101 MHz, 298 K, d_8-THF) spectrum of **8**.

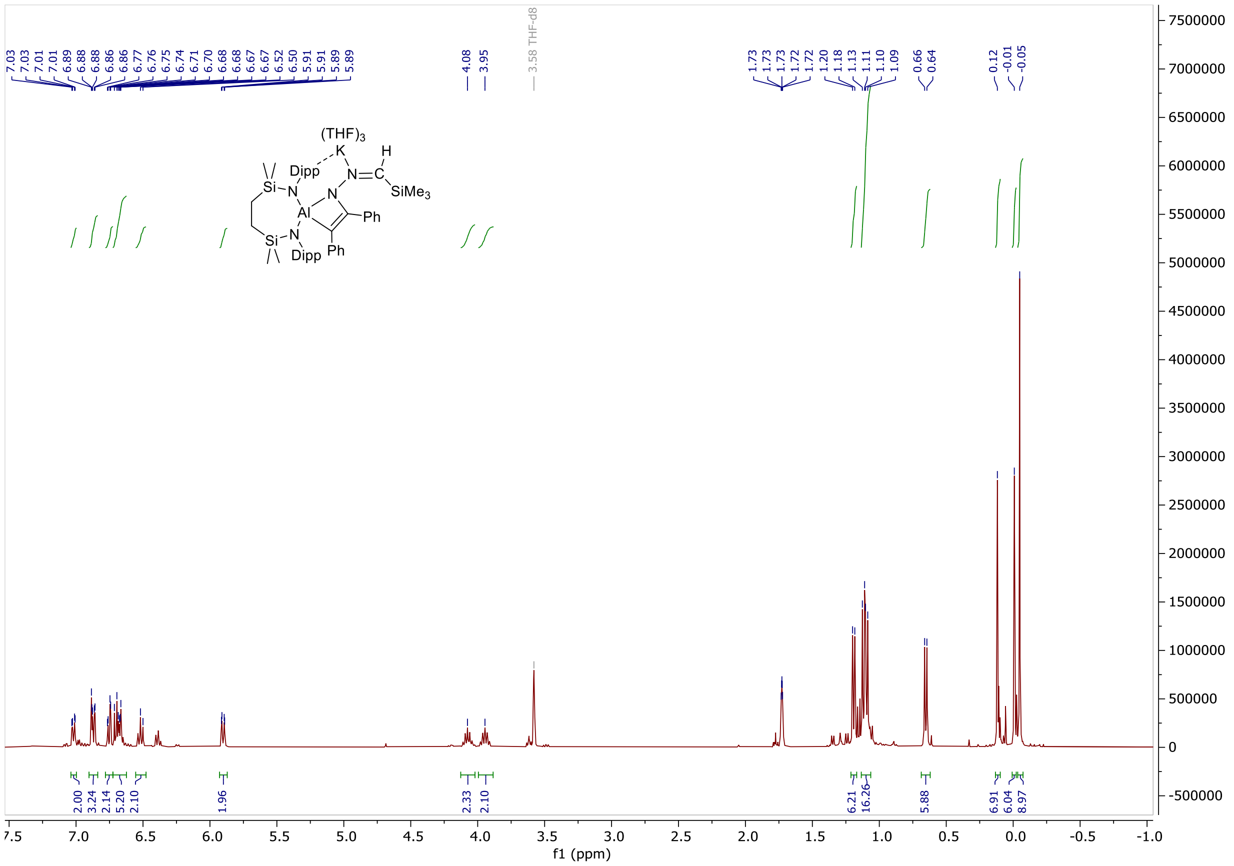

Figure S25. ^{1}H NMR (400 MHz, 298 K, d_8-THF) spectrum of **10**.

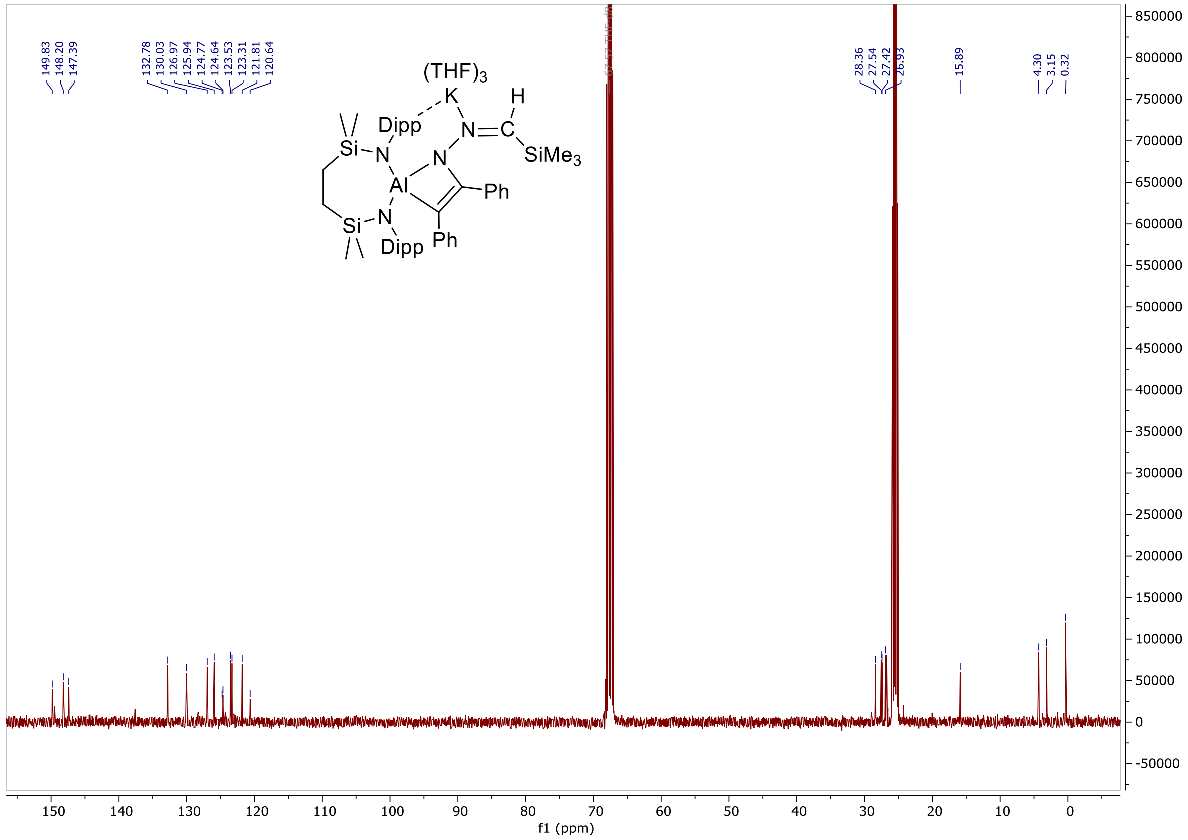

Figure S26. ^{13}C{^{1}H} NMR (101 MHz, 298 K, d_8-THF) spectrum of **10**.

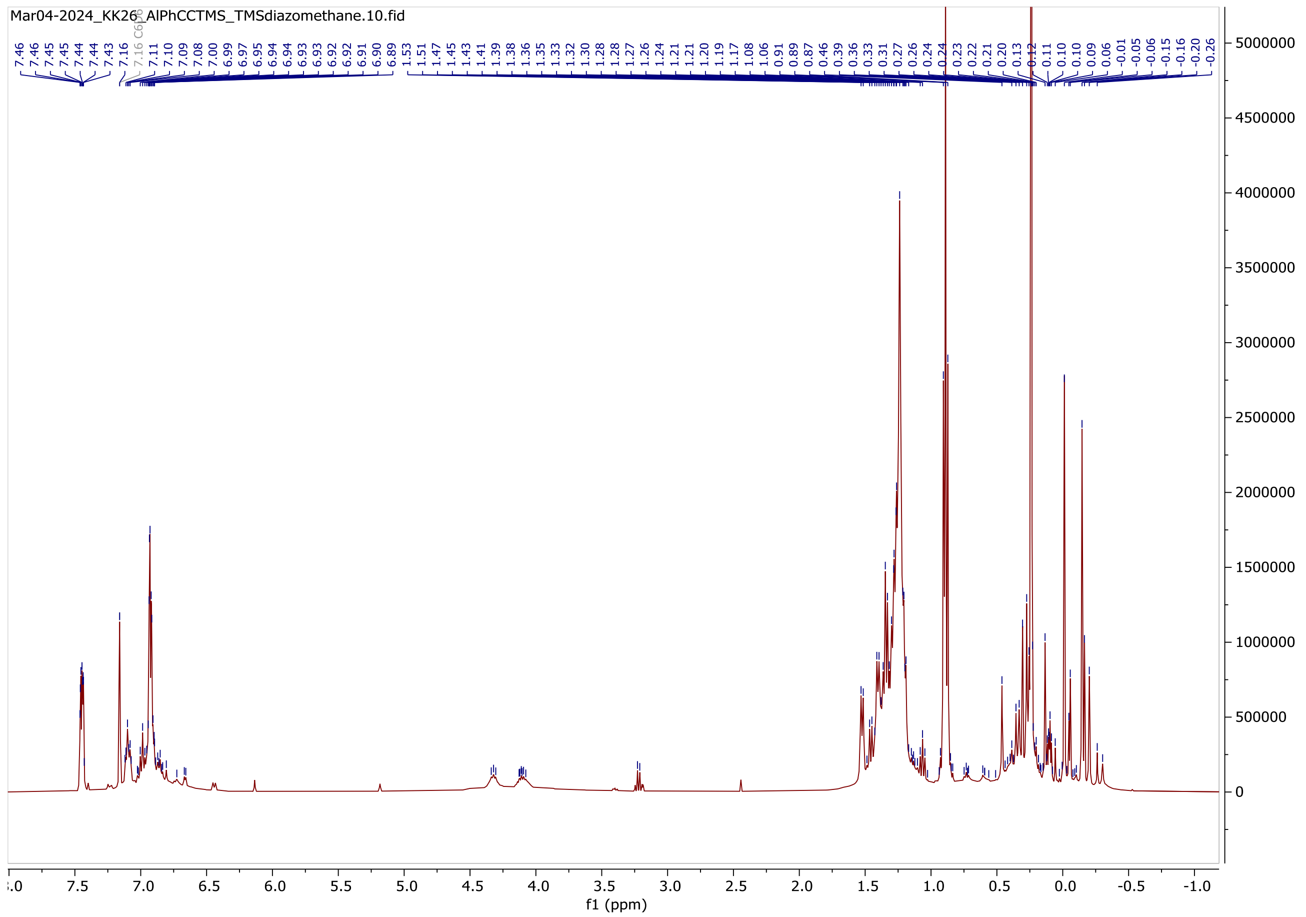

Figure S27. ^{1}H NMR (400 MHz, 298 K, C_6D_6) spectrum resulting from the reaction of **VII** and 2M solution of trimethylsilyl-diazomethane in hexane.

X-ray Diffraction Analysis

Single Crystal X-ray diffraction data for compounds **1**, **2**, **3**, **4**, **6**, **8** and **11** were collected on an Agilent SuperNova EosS2 diffractometer using Cu-Kα (1.54184 Å) radiation, whilst those for compounds **7** and **10** were collected on an Agilent Xcalibur diffractometer using Mo-Kα radiation (λ = 0.71073 Å). In each case, the crystal was maintained at 150 K during data collection. Using Olex2,[1] the structures were solved with the olex2.solve[2] structure solution program or ShelXT and refined with the ShelXL[3] refinement package using Least−Squares minimisation. Where disorder prevailed, distance and ADP restraints were employed (on merit) in disordered regions, to assist convergence.

In **1**, the asymmetric unit contains one molecule of the complex and a region of very disordered, guest, solvent. The latter was broadly identifiable as a THF moiety with unitary occupancy but was ultimately treated using the solvent mask algorithm available in Olex-2. The ligated THF, based on O1, was treated for 50:50 disorder.

The asymmetric unit in **2** contains one molecule of the complex and two molecules of benzene. The solvent was treated for disorder in a 65:45 ratio. The partial occupancy rings were treated as rigid hexagons in the final refinement cycles.

The carbon atoms in the THF ligand based on O1 and all atoms in the comparable ligand containing O2, were treated for 70:30 disorder in the structure of **3**

The asymmetric unit in the structure of **4** constitutes half of a dimer, the remainder of which is generated via the inversion symmetry present in the space group.

The asymmetric unit in **6** comprises a monomer that generates 1-D polymers parallel to the *b*-axis in the gross structure. The carbons in the THF ligand were modelled to take account of 50:50 disorder.

In **7**, the asymmetric unit comprises one molecule of the complex and 1.5 molecules of benzene. The remainder of the half solvent moiety arises via crystallographic inversion symmetry.

The THF ligand based on O3 was modelled to take account of 65:35 disorder in the structure of **8**. Racemic twinning of the sample was addressed in the refinement.

The THF ligands based on O2 and O3 were treated for 65:35 and 70:30 disorders, respectively, in the structure of **10**.

60:40 disorder was modelled for the phenyl group based on C33 and the isopropyl group containing C25 in the structure of **11**, where the asymmetric unit is a monomer. The gross structure is dominated by 1-D polymers parallel to the *b* axis.

Table S1. Crystal Data and Structure Refinement Details for Compounds **1**, **2**, **3**, **4** and **6**.

Identification code	s24msh21 (**1**)	s24msh15 (**2**)	s24msh25 (**3**)	s23msh11 (**4**)	s23msh26 (**6**)
Empirical formula	$C_{64}H_{90}AlKN_2O_3Si_2$	$C_{67}H_{88}AlKN_2Si_3$	$C_{54}H_{90}AlKN_2O_2Si_4$	$C_{49}H_{68}AlKN_2O_3Si_2$	$C_{56}H_{76}AlKN_2O_2Si_2$
Formula weight	1057.63	1071.74	977.71	855.31	931.44
Crystal system	Triclinic	monoclinic	monoclinic	monoclinic	monoclinic
Space group	*P*-1	$P2_1/n$	$P2_1/c$	$P2_1/n$	$P2_1$
a/ Å	14.2337(2)	15.3497(1)	17.7389(2)	19.5884(1)	11.8264(2)
b/ Å	14.7050(2)	26.1030(1)	13.1929(1)	13.7511(1)	19.1808(2)
c/ Å	14.9254(3)	16.3600(1)	25.1734(3)	19.9084(1)	13.1509(2)
α/ °	100.856(1)	90	90	90	90
β/ °	95.001(1)	103.332(1)	94.163(1)	115.597(1)	116.453(2)
γ/ °	90.693(1)	90	90	90	90
U/ Å^3	3055.10(9)	6378.36(7)	5875.73(11)	4836.26(6)	2670.81(8)
Z	2	4	4	4	2
ρ_{calc}/ g cm^{-3}	1.150	1.116	1.105	1.175	1.158
μ/ mm^{-1}	1.612	1.691	1.999	1.924	1.767
F(000)	1144.0	2312.0	2128.0	1840.0	1004.0
Crystal size/ mm^3	0.194 × 0.159 × 0.07	0.151 × 0.134 × 0.091	0.226 × 0.144 × 0.12	0.259 × 0.176 × 0.131	0.117 × 0.099 × 0.047
2θ range for data collection/°	7.752 to 145.79	7.12 to 145.876	7.57 to 145.808	8.098 to 146.142	7.508 to 145.922
Index ranges	-17 ≤ h ≤ 17	-16 ≤ h ≤ 19	-15 ≤ h ≤ 21	-24 ≤ h ≤ 24	-14 ≤ h ≤ 14
	-14 ≤ k ≤ 18	-28 ≤ k ≤ 32	-16 ≤ k ≤ 16	-15 ≤ k ≤ 17	-23 ≤ k ≤ 21
	-18 ≤ l ≤ 18	-20 ≤ l ≤ 19	-28 ≤ l ≤ 31	-24 ≤ l ≤ 24	-14 ≤ l ≤ 16
Reflections collected	64033	88149	32021	64018	29641
Independent reflections, R_{int}	12166, 0.0302	12690, 0.0326	11592, 0.0304	9617, 0.0279	9303, 0.0319
Data/restraints/parameters	12166/173/671	12690/366/742	11592/297/687	9617/0/546	9303/110/626
Goodness–of–fit on F^2	1.048	1.028	1.030	1.028	1.049
Final R1, wR2 [I>=2σ(I)]	0.0348,0.0968	0.0366, 0.1008	0.0431, 0.1123	0.0310, 0.0838	0.0355, 0.0898
Final R1, wR2 [all data]	0.0368, 0.0988	0.0395, 0.1038	0.0539, 0.1216	0.0323, 0.0850	0.0363, 0.0907
Largest diff. peak/hole/ e Å^{-3}	0.55/−0.34	0.32/−0.23	0.45/−0.49	0.36/−0.27	0.32/−0.23
Flack parameter	–	–	–	–	0.006(5)

Table S2. Crystal Data and Structure Refinement Details for Compounds **7**, **8**, **10** and **11**.

Identification code	e24msh08 (**7**)	s23msh12 (**8**)	e23msh05 (**10**)	s24msh26 (**11**)
Empirical formula	$C_{69}H_{90}AlKN_5Si_2$	$C_{65}H_{95}AlKN_5O_3Si_2$	$C_{60}H_{94}AlKN_4O_3Si_3$	$C_{45}H_{74}AlKN_4Si_4$
Formula weight	1111.71	1116.71	1069.74	849.52
Crystal system	triclinic	monoclinic	monoclinic	orthorhombic
Space group	*P*-1	*Cc*	$P2_1/n$	$P2_12_12_1$
a/ Å	12.0778(4)	22.5681(2)	13.6913(2)	11.4974(1)
b/ Å	13.4842(5)	17.7788(1)	24.2224(4)	19.7449(2)
c/ Å	22.4721(8)	16.4956(1)	18.5684(3)	21.8947(3)
α/ °	73.402(3)	90	90	90
β/ °	80.432(3)	103.941(1)	91.5190(10)	90
γ/ °	63.965(4)	90	90	90
U/ Å^3	3147.9(2)	6423.63(8)	6155.79(17)	4970.43(10)
Z	2	4	4	4
ρ_{calc}/ g cm^{-3}	1.173	1.155	1.154	1.135
μ/ mm^{-1}	0.181	1.572	0.204	2.276
F(000)	1198.0	2416.0	2320.0	1840.0
Crystal size/ mm^3	0.366 × 0.261 × 0.231	0.249 × 0.123 × 0.051	0.711 × 0.489 × 0.316	0.189 × 0.131 × 0.096
2θ range for data collection/°	5.902 to 60.662	7.796 to 145.86	5.954 to 57.4	8.076 to 145.864
Index ranges	$-16 \leq h \leq 15$	$-27 \leq h \leq 27$	$-18 \leq h \leq 17$	$-9 \leq h \leq 14$
	$-18 \leq k \leq 17$	$-22 \leq k \leq 15$	$-32 \leq k \leq 30$	$-23 \leq k \leq 24$
	$-31 \leq l \leq 28$	$-20 \leq l \leq 19$	$-25 \leq l \leq 24$	$-27 \leq l \leq 26$
Reflections collected	30231	38488	59765	35764
Independent reflections, R_{int}	15941, 0.0244	12050, 0.0239	15580, 0.0309	9834, 0.0354
Data/restraints/parameters	15941/0/715	12050/154/763	15580/320/754	9834/326/577
Goodness–of–fit on F^2	1.014	1.029	1.019	1.033
Final *R*1, *wR*2 [I>=2σ(I)]	0.0482, 0.1099	0.0360, 0.0964	0.0457, 0.1124	0.0400, 0.1030
Final *R*1, *wR*2 [all data]	0.0674, 0.1212	0.0370, 0.0976	0.0668, 0.1245	0.0422, 0.1053
Largest diff. peak/hole/ e Å^{-3}	0.69/−0.57	0.45/−0.19	0.53/−0.37	0.51/−0.20
Flack parameter	–	0.470(10)	–	0.005(5)

References

1. O. V. Dolomanov, L. J. Bourhis, R. J. Gildea, J. A. K. Howard and H. Puschmann, *J. Appl. Cryst.* 2009, **42**, 339-341.
2. G. M. Sheldrick, *Acta Cryst.* 2015, **A71**, 3-8.
3. G. M. Sheldrick, *Acta Cryst*. 2015, **C71**, 3-8.
